# Phase Equilibria and Crystal Chemistry in Portions of the System SrO-CaO-Bi_2_O_3_-CuO, Part IV— The System CaO-Bi_2_O_3_-CuO

**DOI:** 10.6028/jres.098.034

**Published:** 1993

**Authors:** B. P. Burton, C. J. Rawn, R. S. Roth, N. M. Hwang

**Affiliations:** National Institute of Standards and Technology, Gaithersburg, MD 20899-0001; The Korea Standards Research Institute, Taedok Science Town, Taejon, Chungnam, 305-606 Republic of Korea

**Keywords:** calcium bismuth copper oxide, crystal chemistry, experimental phase relations, phase equilibria

## Abstract

New data are presented on the phase equilibria and crystal chemistry of the binary systems CaO-Bi_2_O_3_ and CaO-CuO and the ternary CaO-Bi_2_O_3_-CuO. Symmetry data and unit cell dimensions based on single crystal and powder x-ray diffraction measurements are reported for several of the binary CaO-Bi_2_O_3_ phases, including corrected compositions for Ca_4_Bi_6_O_13_ and Ca_2_Bi_2_O_5_. The ternary system contains no new ternary phases which can be formed in air at ~700–900 °C.

## 1. Introduction

The discovery of superconductivity in cuprates by Bednorz and Müller [1], and its confirmation by Takagi et al. [2] as being due to the phase La_2−_*_x_*Ba*_x_*CuO_4_, led to a world-wide search for other compounds with higher *T*_c_’s. Identification of the superconducting phase Ba_2_YCu_3_O_6+_*_x_* [3], with a critical temperature *T*_c_ ~90 K [4], has resulted in hundreds of published reports on the properties of this and related phases.

Phases with still higher *T*_c_’s were found in the systems SrO-CaO-Bi_2_O_3_-CuO and BaO-CaO-Tl_2_O_3_-CuO [5,6]. These phases belong mostly to a homologous series A_2_Ca*_n_*_−1_B_2_Cu*_n_*O_2_*_n_*_+4_ (A=Sr, Ba; B = Bi, Tl). In the Bi^+3^ containing systems a phase with *n* =2 and *T*_c_ ~80 K is easily prepared. The exact single-phase region of this phase is not well known, and a structure determination has not been completed because of very strong incommensurate diffraction that is apparently due to a modulation of the Bi positions. Higher *n* (and higher *T*_c_) phases have not been prepared as single-phase bulk specimens (without PbO). We undertook a comprehensive study of phase equilibria and crystal chemistry in the four component system SrO-CaO-Bi_2_O_3_-CuO in the hope that such a study will define the optimum processing parameters for reproducible synthesis of samples with useful properties.

A prerequisite to understanding the phase equilibria in the four component system is adequate definition of the phase relations in the boundary binary and ternary systems. The ternary system SrO-CaO-CuO was the first to be investigated [7,8], followed by the ternary system SrO-Bi_2_O_3_-CuO and its binary subsystems [9,10,11,12]. Preliminary versions have been published of the systems CaO-Bi_2_O_3_-CuO and SrO-CaO-Bi_2_O_3_ [13], and the details of the system SrO-CaO-Bi_2_O_3_ will appear in the near future [14]. The experimental details, phase relations, and crystal chemistry of the binary CaO-Bi_2_O_3_ and the ternary system CaO-Bi_2_O_3_-CuO are the subject of this publication.

In the following discussion of phase equilibria and crystal chemistry, the oxides under consideration will always be given in the order of decreasing ionic radius, largest first, e.g., CaO:1/2Bi_2_O_3_:CuO. The notation 1/2Bi_2_O_3_ is used so as to keep the metal ratios the same as the oxide ratios. The “shorthand” notation is used to designate the phases with C = CaO, B = 1/2Bi_2_O_3_ and Cu=CuO. Thus compositions may be listed simply by numerical ratio e.g., the formula Ca_4_Bi_6_O_13_ can be written as C_2_B_3_ or simply 2:3.

## 2. Experimental Procedures

In general, about 3.5 g specimens of various compositions in binary and ternary combinations were prepared from CaCO_3_, Bi_2_O_3_ and CuO. Neutron activation analyses of the starting materials indicated that the following impurities (in μg/g) were present: in CuO-3.9Cr, 2.8Ba, 28Fe, 410Zn, 0.09Co, 1.9Ag, 0.03Eu, 14Sb; in Bi_2_O_3_-2.1Cr, 0.0002SC, 26Fe, 21Zn, 0.6Co, 0.5Ag, 0.0008Eu, 0.2Sb; in CaCO_3_-1.1Cr, 6Ba, 160Sr, 0.0001Sc, 5Fe, 14Zn, 0.14Co, 0.01Ag, 0.0005Eu, 0.02Sb. The constituent chemicals were weighed on an analytical balance to the nearest 0.0001 g and mixed either dry or with acetone in an agate mortar and pestle. The weighed specimen was pressed into a loose pellet in a stainless steel dye and fired on an MgO single crystal plate, or on Au foil, or on a small sacrificial pellet of its own composition. The pellets were then calcined several times at various temperatures from ~600 to 850 °C, with grinding and repelletizing between each heat treatment. Duration of each heat treatment was generally about 16–20 h. For the final examination a small portion of the calcined specimen was refired at the desired temperature (1–8 times), generally overnight, either as a small pellet or in a small 3 mm diameter Au tube, either sealed or unsealed. Too many heat treatments in the Au tube generally resulted in noticeable loss of Cu and/or Bi.

When phase relations involving partial melting were investigated, specimens were contained in 3 mm diameter Au or Pt tubes and heated in a vertical quench furnace. This furnace was heated by six MoSi_2_ hairpin heating elements with a vertical 4 in diameter ZrO_2_ tube and a 1 in diameter Al_2_O_3_ tube acting as insulators. The temperature was measured separately from the controller at a point within approximately 1 cm of the specimen by a Pt/90Pt10Rh thermocouple, calibrated against the melting points of NaCl (800.5 °C) and Au (1063 °C). After the appropriate heat treatment, the specimen was quenched by being dropped into a Ni crucible, which was cooled by He flowing through a copper tube immersed in liquid N_2_.

In order to approach equilibrium phase boundaries by different synthesis routes, many specimens were prepared from pre-made compounds or two phase mixtures as well as from end members. These were weighed, mixed, and ground in the same way as for the previously described specimens. Also, some specimens were: 1) annealed at temperature (*T*_1_) and analyzed by x-ray powder diffraction; 2) annealed at a higher or lower temperature (*T*_2_) where a different assemblage of phases was observed; 3) returned to *T*_1_ to demonstrate reversal of the reaction(s) between *T*_1_ and *T*_2_. All experimental details are given in Tables 1a and 1b. Phase identification was made by x-ray powder diffraction using a high angle diffractometer with the specimen packed into a cavity 0.127 or 0.254 mm deep in a glass slide. The diffractometer, equipped with a theta compensator slit and a graphite diffracted beam monochromator, was run at 1/4° 2 *θ*/min with Cu*Kα* radiation at 40 KV and 35 MA. The radiation was detected by a scintillation counter and solid state amplifier and recorded on a chart with 1°/2 *θ* = 1 in. For purposes of illustration and publication, the diffraction patterns of selected specimens were collected on a computer-controlled, step scanning goniometer and the results plotted in the form presented.

Equilibrium in this system has proven to be so difficult to obtain that a few specimens were prepared by utilizing lactic acid in an organic precursor route to obtain more intimate mixing at low temperatures [9]. This procedure yielded an essentially single phase amorphous precursor for the composition that contains 66.7 mol % Bi_2_O_3_. At higher Bi contents, pure Bi metal was formed by carbothermic reduction under even the lowest temperature drying procedures in air.

Specimens for solidus and liquidus determinations in the CaO-CuO system were prepared by dissolving mixtures of cupric nitrate and calcium nitrate in distilled water and then drying. The specimens were calcined two or three times between 500 and 700 °C with intermittent grinding. Samples of Ca_1−_*_x_*CuO_2_ were heated in a horizontal tube furnace for 36 to 120 h in air or in oxygen. In determining the *exact* stoichiometry of the compound previously reported as “CaCuO_2_” [7], however, a citrate synthesis route was used [15]. Dried anhydrous calcium carbonate and basic cupric carbonate (Cu(OH)_2_:CuCO_3_) were dissolved in dilute nitric acid and complexed with excess citric acid monohydrate. After drying, the resulting friable, low-density material was calcined at 700 °C either in air or in a flowing oxygen atmosphere until x-ray diffraction revealed the presence of fewer than three phases. It took 18 to 84 h for these synthesis reactions to reach completion.

## 3. Experimental Results and Discussion

Most of the experiments performed on the binary and ternary mixtures of CaO-Bi_2_O_3_-CuO are reported in Table 1a. Additional experiments specifically designed in an attempt to obtain crystals large enough for x-ray single crystal studies are detailed in Table 1b. Crystallographic data for various phases are reported in Table 2.

### 3.1 The System Bi_2_O_3_-CuO

A phase diagram for this system was already published [16], and was redrawn as Fig. 6392 in Phase Diagrams for Ceramists (PDFC) [17]. It apparently contains only one compound Bi_2_CuO_4_, (B_2_Cu). No attempt was made to reinvestigate the melting relations of this system because it does not have any great effect on the phase equilibria of the ternary system with CaO.

### 3.2 The System CaO-CuO

**Fig. 1:**
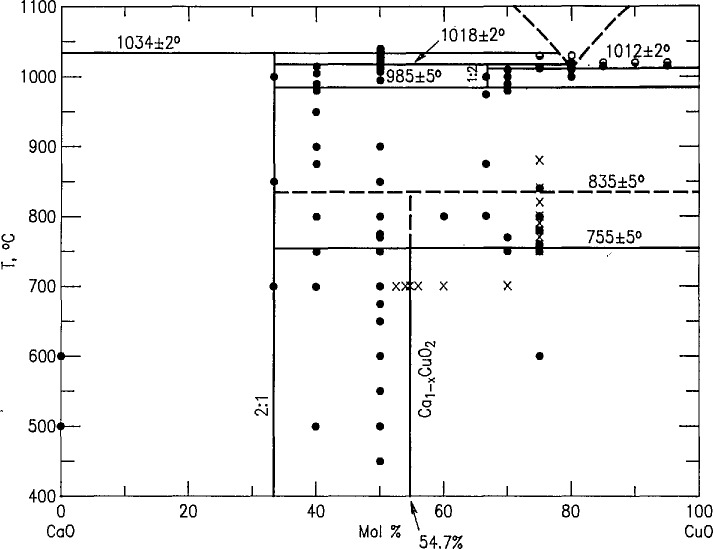
CaO-CuO phase diagram.

Although a revised phase diagram for this system was previously reported [7], further experimental evidence (Table 1a) was accumulated in this study and the diagram was revised again [18] as shown in Fig. 1. The CaCu_2_O_3_ compound, which was reported to be stable only above 950 °C [19], was found to be stable between 985 and 1018 °C. Previously determined temperatures, 1020 and 1013 °C [20,7] for the decomposition of CaCu_2_O_3_(CCu_2_) and for eutectic melting, respectively, are within experimental error of the new values, 1018 ± 2 °C and 1012 ± 2 °C.

#### 3.2.1 Ca_2_CuO_3_

The Ca_2_CuO_3_(C_2_Cu) compound decomposes into CaO plus liquid above 1034 ± 2 °C, which is slightly above the previous estimate of 1030 °C [20,7]. The composition of the eutectic reaction is 20CaO−80CuO±5%, as determined from the presence or absence of the Ca_2_CuO_3_ phase in samples of varying compositions that were quenched from 1020 °C.

#### 3.2.2 Ca_1_*_−x_*CuO_2_

**Fig. 2:**
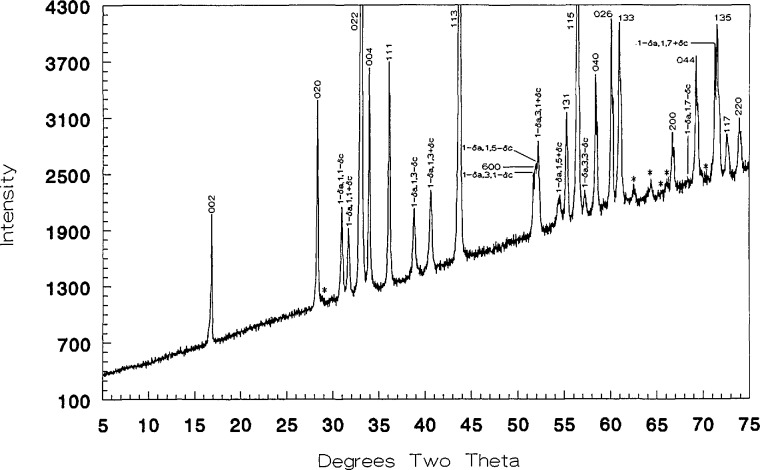
Ca_1−_*_x_*CuO_2_ x-ray diffraction powder pattern (CaO:CuO 45.328:54.672).

Samples prepared with an original Ca:Cu ratio of 45.33:54.67 contained no detectable CaO or CuO after heating in oxygen at 700 °C, as demonstrated by x-ray diffraction (Fig. 2 and Table 3). Compositions with original Ca:Cu ratios of 45.20:54.80 and 45.45:54.54 (≈5:6) yielded x-ray patterns which indicated the presence of excess CuO and excess CaO, respectively. Therefore, the Ca:Cu ratio for this compound is 0.453:0.547 or Ca_1_*_−x_*CuO_2_ with the composition Ca_0.828_CuO_2_ (*x* = 0.172) at 700 °C in oxygen. The single phase region for this phase probably varies with temperature and partial pressure of oxygen. The composition and structural analyses of this phase have been recently reported [15]. The x-ray powder diffraction pattern for Ca_1−_*_x_*CuO_2_ is shown in Fig. 2 and the indexed data is given in Table 3. This compound decomposes into Ca_2_CuO_3_ plus CuO above 755 °C in air and 835 °C in oxygen. In Fig. 1, the experiments conducted in air and those conducted in an oxygen atmosphere are indicated by the dashed line and the crosses, respectively. At 675 °C, Ca_1−_*_x_*CuO_2_ can be synthesized from CaCO_3_ plus CuO but the run product never fully equilibrates to a single- or two-phase assemblage. Rather, the metastable three-phase assemblage Ca_1−_*_x_*CuO_2_+CaO+CuO persists: after five cycles of heating with intermittent grinding the relative proportions of phases were Ca_1−_*_x_*xCuO_2_>CaO> CuO and they remained that way for an additional overnight heat treatments. Because of its greattional 31 overnight heat treatments. Because of its great persistence, Ca_1−_*_x_*CuO_2_ is interpreted as being an equilibrium phase, but it should be noted that reversal of its decomposition (synthesis from CuO+Ca_2_CuO_3_) was not successfully demonstrated.

#### 3.2.3 Cu_2_O in the Binary System

Cu_2_O, which is known to be stable in air only above 1026 °C, was found in this system above 1012 °C. Therefore, Cu^+^ and Cu^2+^ must have coexisted in the samples that were quenched in air from temperatures between 1012 and 1026 °C. The Cu_2_O observed in samples that were quenched from below 1026 °C is probably formed during solidification of the liquid phase; i.e., an oxygen deficiency in the liquid may result in the solidification of Cu_2_O as well as CuO.

### 3.3 The System CaO-Bi_2_O_3_

**Fig. 3:**
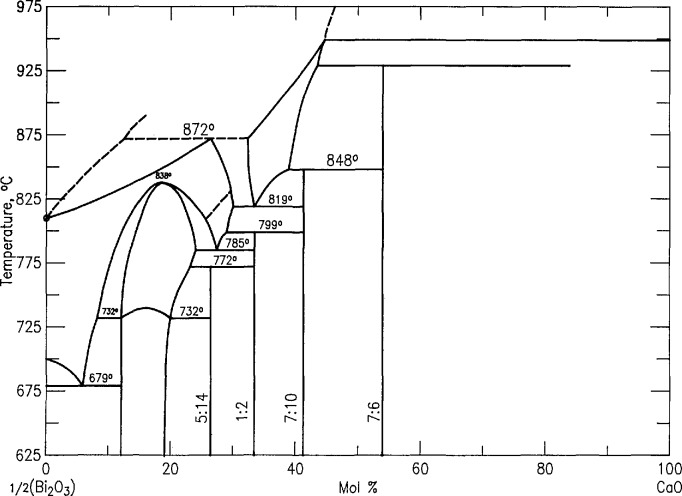
CaO-1/2Bi_2_O_3_ phase diagram as changed from PDFC 6380-Conflant et al.

**Fig. 4:**
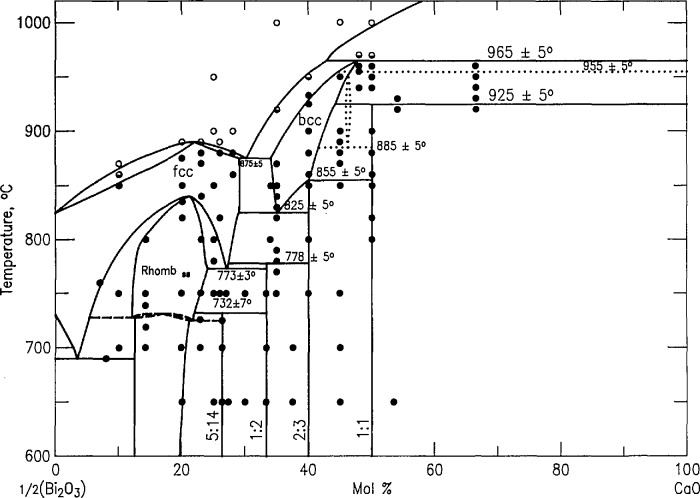
CaO-1/2Bi_2_O_3_—present phase diagram.

The phase equilibria diagram for the system CaO-Bi_2_O_3_ was reported in [21] and redrawn as Fig. 6380 in PDFC [17]. It is reproduced here as Fig. 3 with the scale changed to l/2Bi_2_O_3_-CaO instead of Bi_2_O_3_-CaO, to maintain consistency with the other phase diagrams in this report. An interpretation of the experimental results recorded in Table 1 was published in [19] and it is shown in Fig. 4 (cf. Fig. 3). The major differences between our new diagram and the one presented in [21] are: 1) the composition of “Ca_7_Bi_10_O_22_” [21,22] is revised to Ca_4_Bi_6_O_13_ (2:3) and its crystal structure is reported in [23]; 2) the composition of “Ca_7_Bi_6_O_16_” [21,22] is now reported as Ca_2_Bi_2_O_5_, and its crystal structure is given in [24]; 3) a metastable phase ~Ca_6_Bi_7_O_16.5_ was formed at about 925 °C on the CaO-rich side of Ca_2_Bi_2_O_5_, but at about 885 °C on the CaO-poor side; 4) melting relations have been determined in the region of 20–50 mol *%* CaO.

#### 3.3.1. Rhombohedral Solid Solution (Sillen Phase-Rhomb)

The rhombohedral solid solution was first reported by Sillen [25]. Phase relations in the CaO-rich region of the Sillen phase field were previously [20] represented as exhibiting a congruent transition to the fcc solid solution, and the present experiments indicate such a point at (~22 mol % CaO, ~835 °C). Conflant et al. [21] reported a phase transition from one rhombohedral phase to another at about 735–740 °C. Differential thermal analysis of a 1:6 ratio CaO:1/2Bi_2_O_3_ specimen confirms the presence of a reversible transition at about 735 °C. Samples quenched from ~750 °C are clearly rhombohedral as previously reported [21,22], but x-ray patterns (Figs. 5a, 5b; Tables 4, 5, 6) from samples that were quenched from ⩽735 °C exhibit peak splitting and faint superstructure reflections (Fig. 5b). The diffraction patterns for both the high and low temperature forms are much sharper if the specimens are not ground after quenching. Apparently, it is easy to induce mechanical deformation in these samples by grinding. The peak splitting can be indexed with an orthorhombic cell *a* =6.8188(3), *b* =3.9531(2), and *c* =27.830(1) Å, which is most easily observed in the rhombohedral (0,2,13) and (3,0,9) reflections corresponding to (2,2,13)+ (4,0,13) and (3,3,9)+ (6,0,9), respectively, in the orthorhombic indexing (Figs. 5a, 5b, and Tables 5, 6). Dimensionally the unit cell is orthorhombic, but the symmetry cannot be higher than monoclinic because it is the derivative of a rhombohedral (rather than hexagonal) high symmetry phase. Single crystals prepared at 700 °C with a salt eutectic flux (Table 1b) give a biaxial interference figure, in polarized light, parallel to the pseudo-rhombohedral *c* axis.

#### 3.3.2. “Face-Centered-Cubic” Solid Solution (“fcc”)

**Fig. 6:**
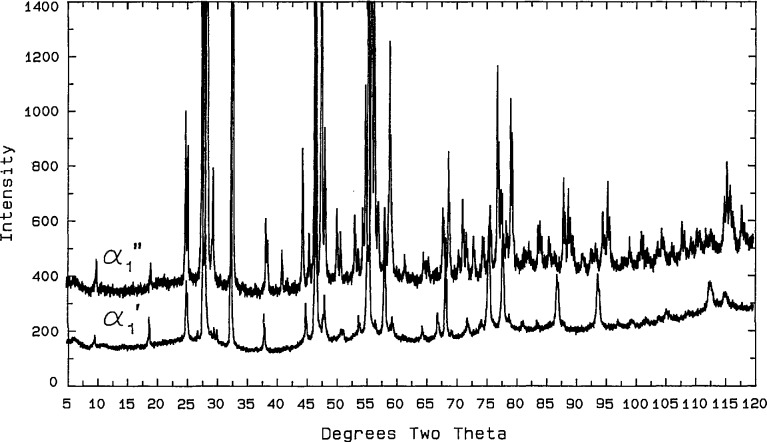
x-ray powder diffraction pattern of the fcc phase showing splitting and superstructure of *α*_1_′ and *α*_1_*″.*

**Fig. 7:**
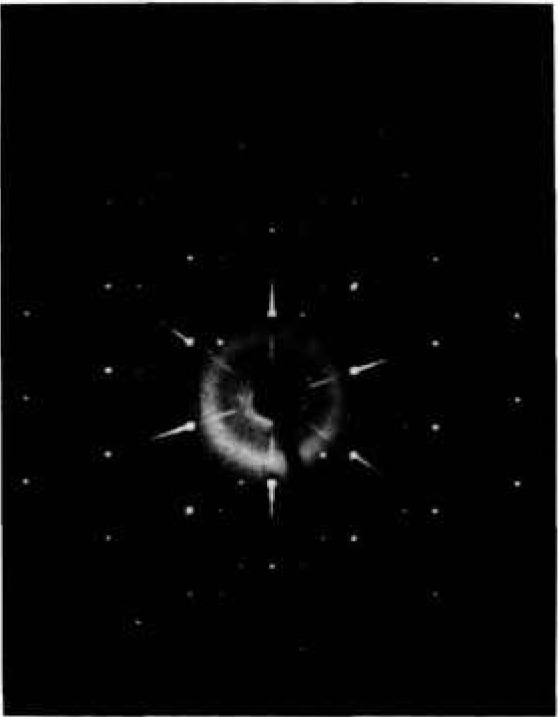
x-ray precession photograph of the fcc α_1_*″* phase (Mo radiation).

Levin and Roth [26] demonstrated that the solidus temperature of fcc Bi_2_O_3_ (*α*_1_ in [21]) increases with additions of CaO. Conflant et al. [21] depicted its homogeneity range as extending to temperatures above the rhombohedral Sillen phase, and they did not include a congruent melting point. The present work and [18], however, indicate that there is a congruent melting point between 20 and 23 mol % CaO at about 885 °C. The phase diagram in [21] includes a dashed line which defines a small *α*_1_′ region in the CaO-rich, low temperature portion of the fcc field. Present results are essentially in agreement with this finding; i.e., all x-ray diffraction patterns from quenched “fcc” samples that contain at least 20 mol % CaO exhibit the superstructure peaks described in [21] plus a very slight splitting of cubic diffraction maxima that was not described in [21] (Fig. 6, Table 7). The observed splitting of substructure peaks of α_1_′ fits rhombohedral symmetry with *a*_H_=7.7427(9), *c*_H_ = 9.465(1) Å, *c/a* = 1.2224. The complete field, extending to about 30 mol % CaO, is labeled “fcc” because neither the data presented here nor that in [20] provides a sound basis for drawing definitive phase boundaries. The minimum shown in Fig. 4 at ~773 °C for the CaO-rich end of this solid solution is in relatively good agreement with the value of 785 °C which can be interpreted from [21] (Fig. 3). When a single-phase specimen of composition near this minimum (5:14-3:8, CaO:1/2Bi_2_O_3_) is quenched after 10 min annealing at ~760 °C (~13 °C below the equilibrium minimum), the rhombohedral splitting of cubic maxima was greatly enhanced; this is the *α*_1_″ phase of [21] (Fig. 6; Table 8). As with the rhombohedral Sillen-type phases, these rhombohedrally distorted fcc phases are highly susceptible to mechanical damage during routine grinding, therefore the line splitting of α_1_’ can only be seen if the quenched specimen is not ground. X-ray analysis of this sample yielded *a*_H_ = 7.616, *c*_H_ = 9.6477, *c/a* =1.2668, whereas hexagonal indexing of a truly cubic pattern would give *c/a*= 1.2247; [1,1,1]*_c_*=[0,0,0,3]_H_ and ${\left[2,2,0\right]}_{c}={\left[2,2,4\;\bar{4},0\right]}_{H}$. Thus, the rhombohedrally distorted phase that was quenched from the stable “fcc” region (*α*_1_′) had a *c/a* ratio that was slightly smaller than the cubic value, but the metastable lower-temperature phase (*α*_1_″) that was quenched from below the “fcc” region had a *c/a* ratio that was considerably larger than the cubic value. Single crystal x-ray precession patterns from the *α*_1_″ phase (Fig. 7) can be indexed with either a monoclinic or a rhombohedral cell with *a* =4*a*_sub_ as shown in Table 8.

#### 3.3.3. The “Body-Centered-Cubic” Solid Solution (“bcc”)

**Fig. 8:**
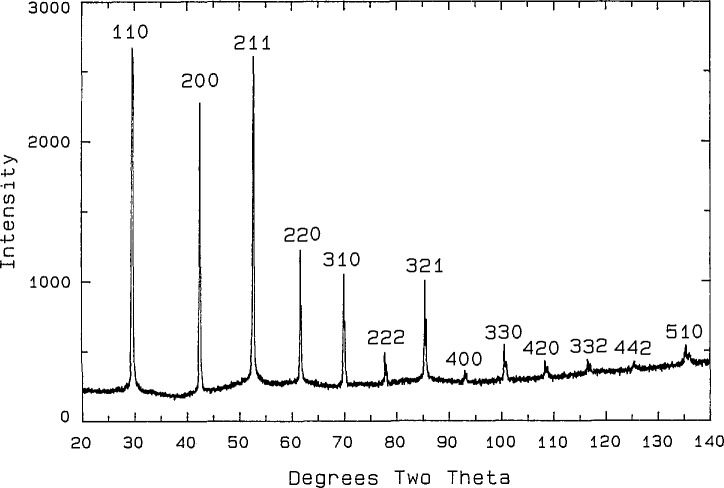
x-ray powder diffraction pattern for the bcc phase.

**Fig. 9:**
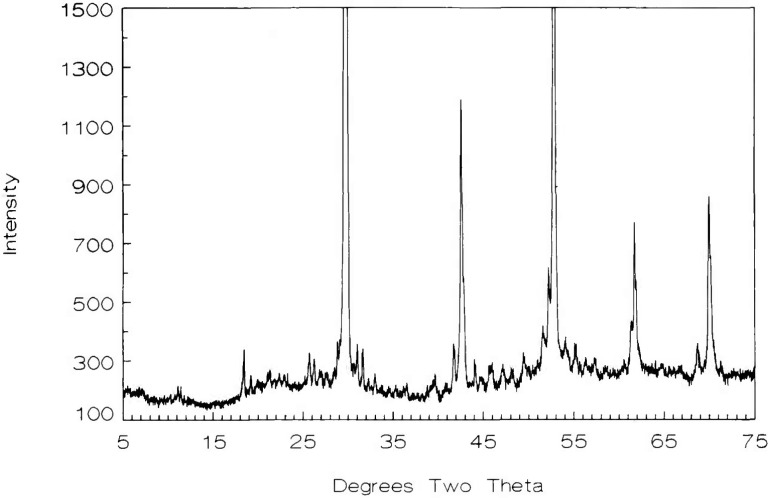
x-ray powder diffraction pattern for the distorted bcc phase with line splitting and superstructure (CaO:1/2Bi_2_O_3_ 2:3 860 °C).

**Fig. 10:**
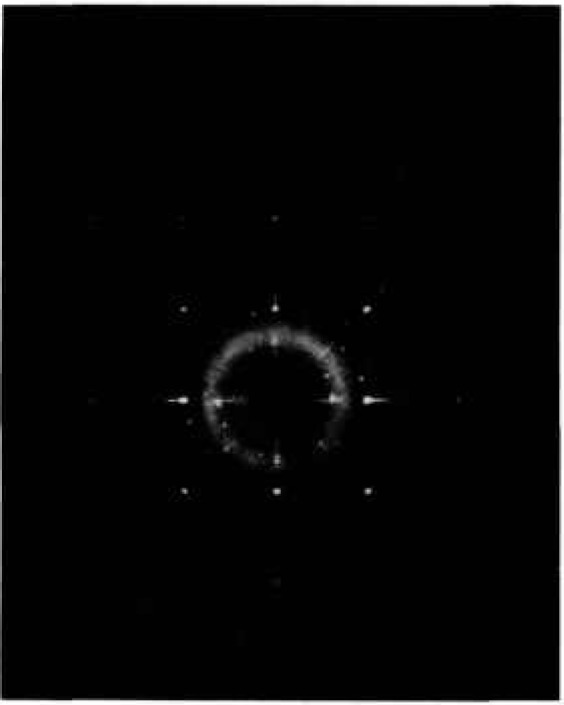
x-ray precession photograph of the bcc distorted phase (Mo radiation).

The phase referred to as body-centered-cubic (“bcc”) solid solution was reported as a high temperature phase in [21]. In the present study this phase was found to extend from about 35 to 45 mol *%* CaO. The exact boundaries of the two-phase “fcc-bcc” region were not determined because the compositions of coexisting phases were not consistently reproduced. Just as with the “fcc” phase the “bcc” phase also exhibits line splitting and superstructure. Distortions from cubic symmetry (Fig. 8, Table 9), seem to be greatest in samples that are quenched from the region near the decomposition point of the 2:3 phase, (Fig. 9, Table 10). Single crystal x-ray diffraction precession data (Fig. 10) confirm the distortion recorded in Fig. 9 and Table 10 and indicate the nature of the superstructure. CaO-rich phase boundaries of the “bcc” field have not been precisely determined in part because of complications arising from the presence in many experiments of a metastable phase (see “C-mon” below). This bcc-type phase was found to be stable down to a minimum temperature of 825 ± 5 °C (Fig. 4) which is in good agreement with the value of 819 °C interpreted from [20] (see Fig. 3).

#### 3.3.4. “Ca_5_Bi_14_O_26_” (C_5_B_14_-5:14)

**Fig. 11:**
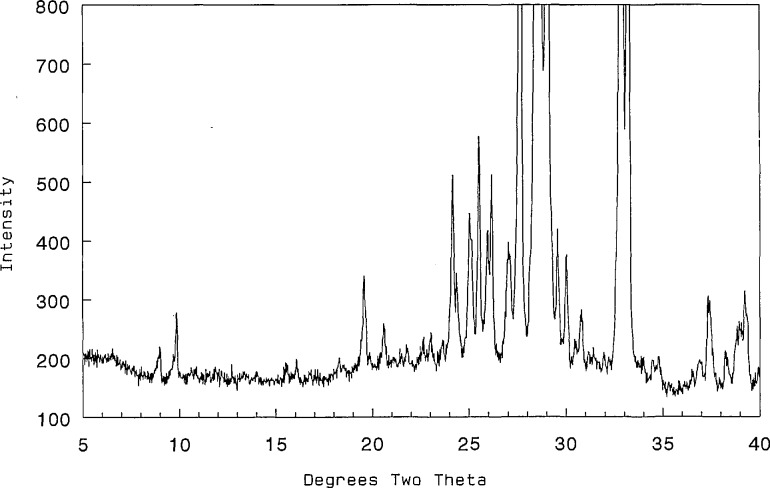
X-ray powder diffraction pattern for the Ca_5_Bi_14_O_26_ compound.

**Fig. 12:**
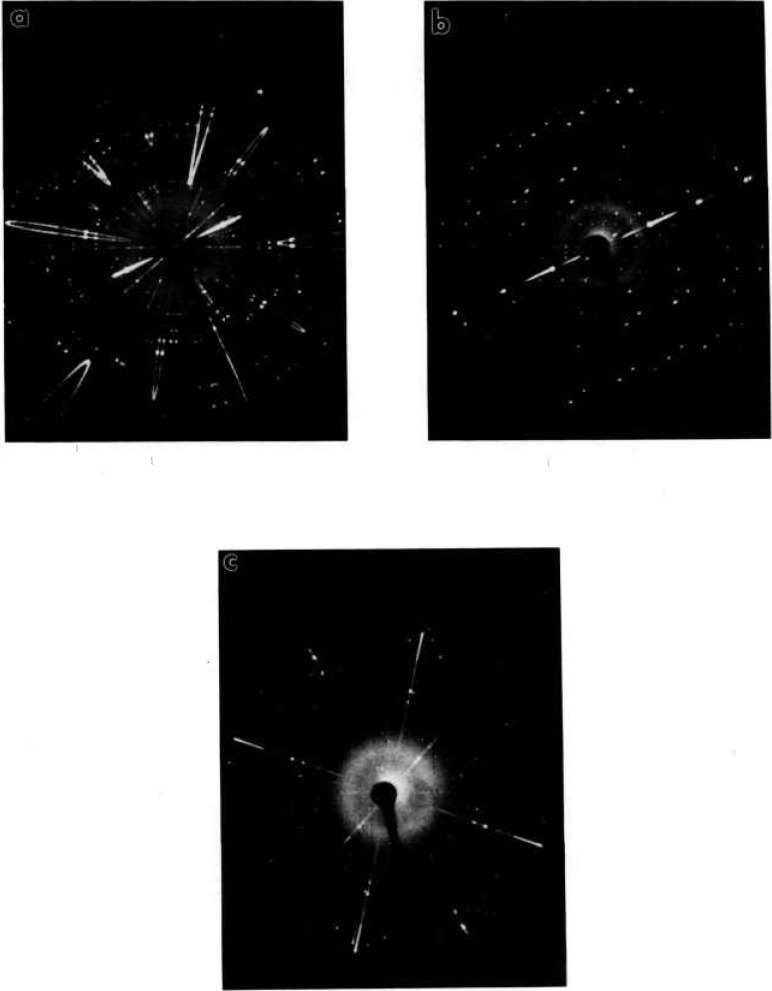
**X**-ray precession photographs of Ca_5_Bi_14_O_26_ (Mo radiation) (a) (*hOl*) unfiltered μ = 10°, (b) (*hOl*) Zr filter (c) alternate plane, unfiltered.

A compound with the composition Ca_5_Bi_14_O_26_ was previously reported [21,22] as stable up to at least 650 °C. We have no contrary evidence and indeed an apparently single phase x-ray diffraction pattern can be obtained for the 5:14 ratio (26.32% CaO; Fig. 11, Table 11) by annealing a quenched liquid of this composition overnight at 650 °C. The exact composition should be regarded as provisional, however, pending a crystal structure determination. The x-ray pattern in Table 11 corresponds well with that published in [22] except for a small but consistent shift in observed *d* amounting to ~1/4° 2*θ* for CuK*α* radiation. Apparently the earlier work had an unrecognized deviation in calibration of the diffraction data. The diffraction pattern has not yet been indexed even with the aid of some single crystal data (Fig. 12). The complexity of the pattern and consideration of the single crystal data suggests tri-clinic symmetry.

At 732 ± 7°C the 5:14 phase decomposes to a mixture of the rhombohedral phase plus CaBi_2_O_4_ (1:2). This equilibrium was demonstrated by both the breakdown of single phase material after heating above this range, and by nucleation of 5:14 in a two phase mixture of rhombohedral + 1:2 below it. This is considerably lower than the value of 772 °C which may be interpreted from [21] (Fig. 3).

#### 3.3.5. CaBi_2_O_4_ (CB_2_-1:2)

**Fig. 14:**
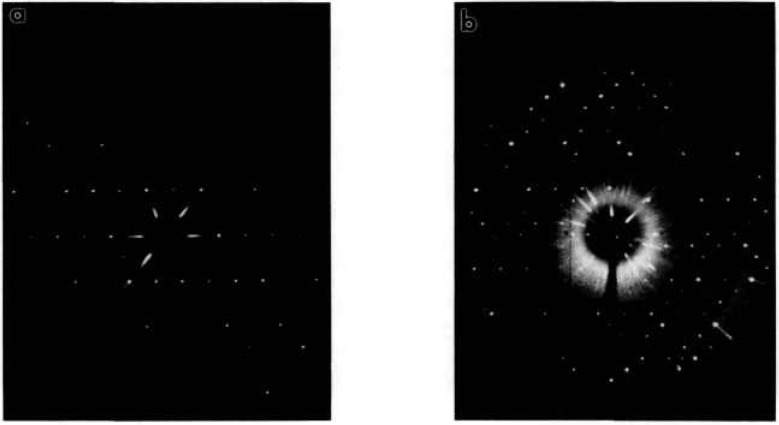
X-ray precession photographs of CaBi_2_O_4_ (Mo radiation) (a) (*h0l*), (b) (*hll*).

The compound CaBi_2_O_4_ was synthesized at 650 °C [22] and reported as stable up to about 800 °C [21] where it was shown (Fig. 3) to decompose to fcc plus 2:3. Apparently inconsistent data in our own work required us to determine the decomposition temperature by simultaneous quenching of single phase 1:2, originally prepared by annealing at 650 °C, and reheating a sample of quenched liquid from which fcc plus 2:3 was synthesized. These experiments suggest that the 1:2 phase is not stable above 778 ± 5 °C. This may be compared with the value of 799 °C which can be interpreted from [21] (Fig. 3). The 1:2 phase often occurs along with other phases in samples that are air quenched from temperatures greater than about 800 °C. The x-ray powder diffraction pattern of the 1:2 phase Fig. 13, Table 12, corresponds well with that reported in [22] except for the observed shift in 2 *θ* mentioned in section 3.3.4. Several attempts were made to synthesize single crystals of the 1:2 phase (see Table 1b), but the only procedure that succeeded was to anneal single phase 1:2 + a 50/50 NaCl/KCl flux (50/50 flux/charge) at 775 °C and then cool at 1 °C/h to 645 °C. The single crystal x-ray diffraction precession data are shown in Fig. 14. The x-ray powder diffraction pattern was indexed on the C-centered monoclinic cell C2/*c* obtained from the single-crystal precession data. The lattice parameters refined by least-squares analysis with the aid of calculated structure factors and the calculated powder pattern based on single crystal structure determination are *a* = 16.6295(8), *b* = 11.5966(5), *c* = 14.0055(6) Å, and *β* = 134.036(3)°.

#### 3.3.6. Ca_4_Bi_6_O_13_ (C_2_B_3_-2:3)

**Fig. 15:**
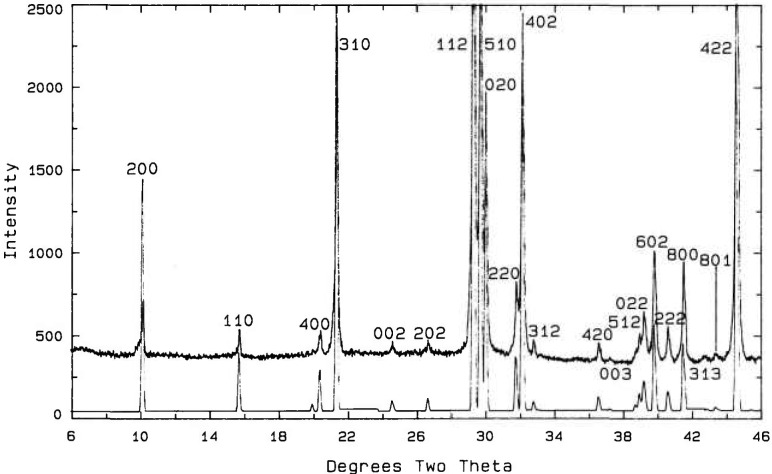
X-ray powder diffraction pattern of the Ca_4_Bi_6_O_13_ compound.

**Fig. 16:**
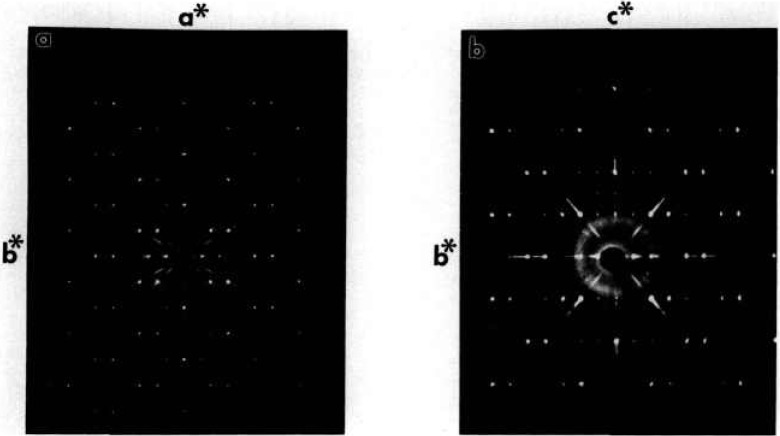
X-ray precession photographs of Ca_4_Bi_6_O_13_ (Mo radiation), (a) *(hk0*), (b) (*0ld*).

The compound “Ca_7_Bi_10_O_22_”, (41.176 mol % CaO) was reported in [22] and [21], and the phase diagram shown in [21] can be interpreted as indicating that it decomposes at about 848 °C. (Fig. 3 in [20]). Experiments performed in the present work (Table 1) indicate that the composition of this phase is really 2:3 (40 mol *%* CaO) rather than 7:10, but the decomposition temperature (Table 1 and Fig. 4) of 855 ±5 °C is in good agreement with [21]. The x-ray powder diffraction pattern of this phase is shown in Fig. 15 and recorded in Table 13. These results agree well with those in [22] (except for the shift in 2 *θ* previously mentioned). Single crystals of Ca_4_Bi_6_O_13_ were grown both by utilizing a 50/50 NaCl/KCl flux and by reannealing a quenched liquid. The compound is orthorhombic *a* =17.3795(5), *b* =5.9419(2), *c* =7.2306(2) Å, with a C-centered space group, as determined from single crystal x-ray precession photographs Fig. 16) and x-ray diffraction datarefined by least squares. A complete crystal structure determination [23] including single crystal x-ray analysis, neutron diffraction Rietveld analyses, and measurements of second harmonic generation, proved that the true space group is the non-centrosymetric C2mm. The crystal structure was reported in [23] from data collected on crystals prepared in this study. A complete discussion of the indexing of this phase with comparison to the calculated powder pattern is given in [27]. The crystal structure determination [23] reveals that Bi^+3^ occurs in two coordination types with 2/3 of the Bi^+3^ ions five-coordinate and 1/3 of the Bi^+3^ ions only three-coordinate, by oxygen. Determinations of the crystal structures of more of these phases will perhaps result in a better understanding of the role played by Bi^3+^ coordination in 3- and 4-component superconductors.

#### 3.3.7. Ca_2_Bi_2_O_5_ (C_2_B_2_-1:1)

**Fig. 17:**
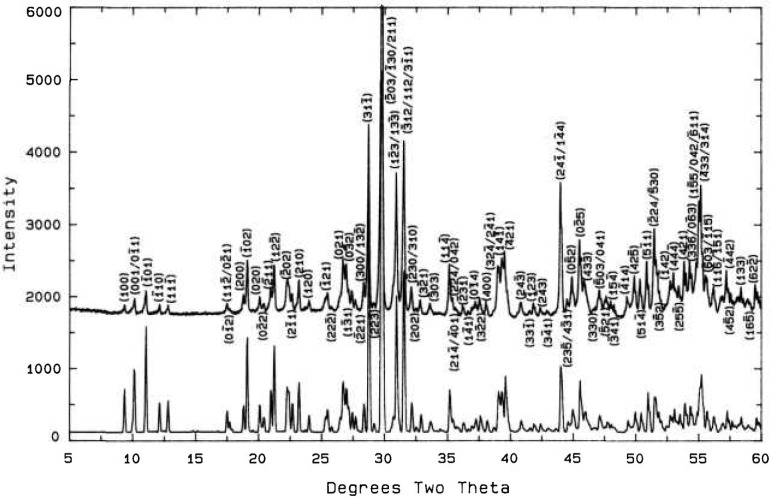
X-ray powder diffraction pattern of the Ca_2_Bi_2_O_5_ compound.

**Fig. 18:**
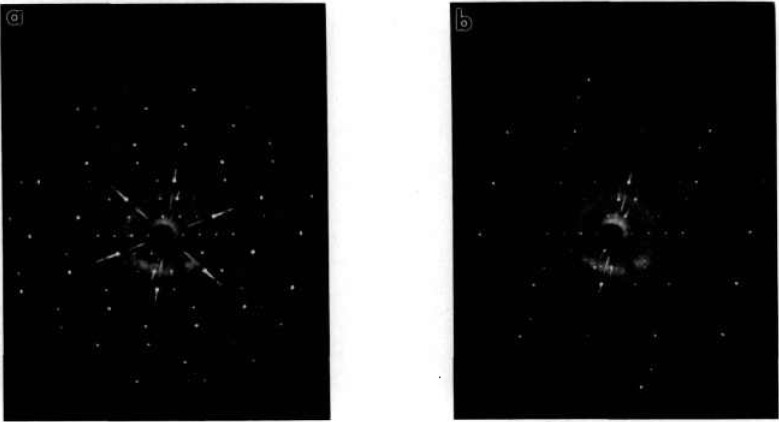
X-ray precession photographs of Ca_2_Bi_2_O_5_ (Mo radiation) (a) (*hk0*), (b) (*h0l*).

The compound “Ca_7_Bi_6_O_16_”, (53.846 mol *%* CaO) was reported in [22] and [21], and the phase diagram in [21] (redrawn as Fig. 3) can be interpreted as indicating that it decomposes at about 929 °C. Experiments performed in the present work (Table 1) combined with a structure determination performed on crystals prepared in this study [24] indicate that the composition of this phase is really 1:1 (50 mol % CaO) rather than 7:6. The x-ray powder diffraction pattern of the phase shown in Fig. 17 and Table 14 agrees well with that reported in [22] (except for the shift in 2 *θ* noted above). Single crystal x-ray diffraction precession photographs (Fig. 18) indicate that the 1:1 compound is triclinic, and powder x-ray diffraction data [27] yield least squared values of *a* = 10.1222(7), *b* = 10.146(6), *c* = 10.4833(7) Å, *α* = 116.912(5), *β* = 107.135(6), *γ* = 92.939(6)°. The indexing of this pattern out to high angles in 2 *θ* could only be accomplished with the aid of calculated structure factors and the calculated powder pattern based on the single crystal structure determination reported in [24]. The structure determination reveals a unique Bi^+3^ coordination of U-shaped Bi_3_O_11_ groups with one five-fold coordinated Bi^+3^ bridging two four-fold “saw-horse” shaped polyhedra [24].

#### 3.3.8 “C-mon” MetastablePhase ~Ca_6+_*_x_*Sr_6__*_x_*Bi_14_O_33_ (*x* → 6)

**Fig. 19:**
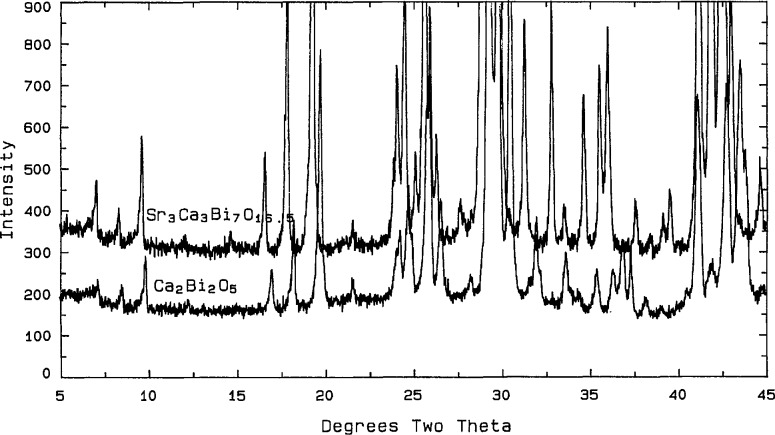
X-ray powder diffraction pattern comparing the “C-mon” metastable phase ~ Ca_6+_*_x_*Sr_6−_*_x_*Bi_14_O_33_ *x* → 6 to the ternary *x* → 0.

When the 1:1 phase is heated between 885 and 925 °C for 20 min to 3 h a metastable C-centered monoclinic phase is formed which may be nearly single phase [a = 21.295(4), *b* =4.3863(8), *c* =12.671(2) Å, and *β* = 102.74(1)°]. After overnight heat treatments, however, this phase decomposes to a “bcc” plus CaO assemblage. Comparison of the X-ray powder diffraction patterns (Fig. 19, Table 15) for this phase and for Ca_6+_*_x_*Sr_6−_*_x_*Bi_14_O_33_ (*x* ~ 4.8) indicates that it is the metastable end member extension of the stable ternary solid solution.

### 3.4 The System CaO-Bi_2_O_3_-CuO

**Fig. 20:**
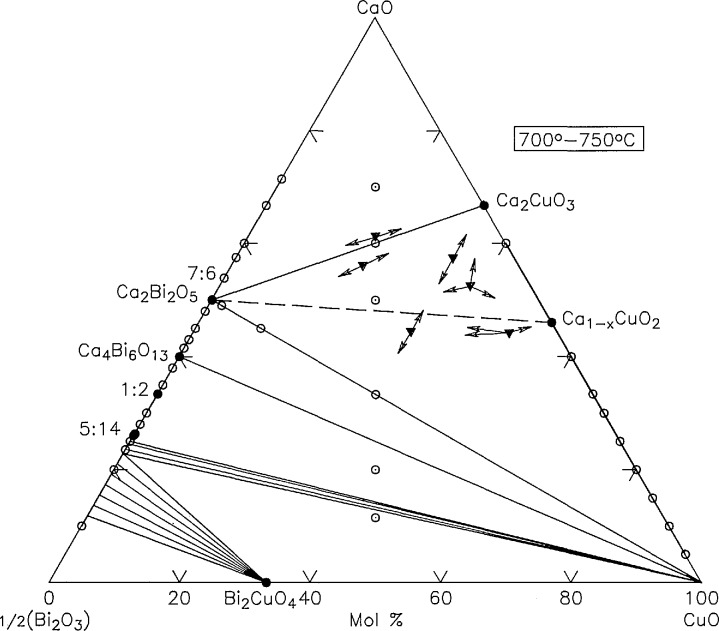
CaO-Bi_2_O_3_-CuO 700–750 °C phase diagram.

**Fig. 21:**
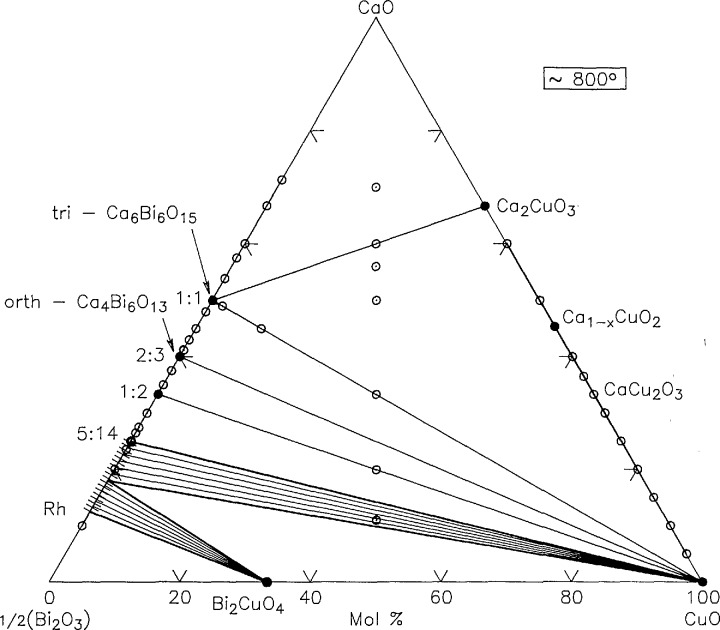
CaO-Bi_2_O_3_-CuO 750–800 °C phase diagram.

Ternary phase relations of the system CaO-l/2Bi_2_O_3_-CuO have been studied at temperatures between 700 and 900 °C. No ternary compounds were discovered, but new data on the CaO-l/2Bi_2_O_3_ and CaO-CuO binaries have been incorporated. The ternary phase relations at 700–750 and 750–800 °C are shown in Figs. 20 and 21 respectively. There remains some uncertainty about the equilibrium phase relations involving Ca_1−_*_x_*CuO_2_.

To verify that the three-phase equilibria inferred from synthesis runs (products of a synthesis from CaCO_3_, Bi_2_O_3_, and CuO) reflected equilibrium phase assemblages, various three phase mixtures of pre-made binary compounds were reacted isothermally. For example, such experiments demonstrate that a mechanical mixture of Ca_4_Bi_6_O_13_+7Ca_2_CuO_3_+3Ca_4_._533_Cu_5.467_O_10_ (bulk composition 51.80: 9.84: 38.36) is metastable with respect to a mixture of Ca_2_Bi_2_O_5_+Ca_2_CuO_3_+Ca_4_._533_Cu_5.467_O_10_ at 700 °C. Because the nucleation (or increase in volume fraction) of Ca_1−_*_x_*xCuO_2_ from binary compounds was never demonstrated at 700 °C (see Sec. 3.2.2) the possibilities of three phase equilibria including Ca_2_CuO_3_ (and/or Ca_1−_*_x_*CuO_2_) plus Bi_6_Ca_4_O_13_ can not be ruled out. For example, the mechanical mixture 5Ca_2_CuO_3_+Ca_4_Bi_6_O_13_ which has a bulk composition of 56:24:20 shows no convincing evidence of Ca_1−_*_x_*CuO_2_ even after six heating/grinding treatments at 700 °C.

## 4. Summary

A new phase diagram is presented for the system CaO-CuO with the composition of the phase Ca_1−_*_x_*CuO_2_ corresponding to a Ca:Cu ratio of 45.33: 54.67. This compound decomposes at ~ 755 °C in air and 835 °C in O_2_. The phases previously reported as “Ca_7_Bi_10_O_20_” and “Ca_7_Bi_6_O_16_” [21,22] are really Ca_4_Bi_6_O_13_ and Ca_2_Bi_2_O_5_ respectively. X-ray powder and single crystal data are reported for almost all of the binary phases encountered. No ternary phases were found in the system CaO-l/2Bi_2_O_3_-CuO. Above 775 °C CuO is in equilibrium with all of the binary CaO-Bi_2_O_3_ phases, and this is probably true below 775 °C as well.

## Figures and Tables

**Fig. 5a:**
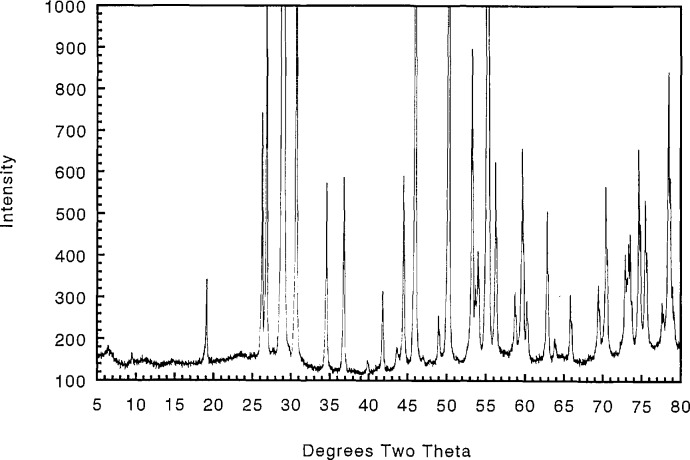
x-ray powder diffraction pattern CaO:1/2Bi_2_O_3_ 1:6 quenched from 740 °C.

**Fig. 5b:**
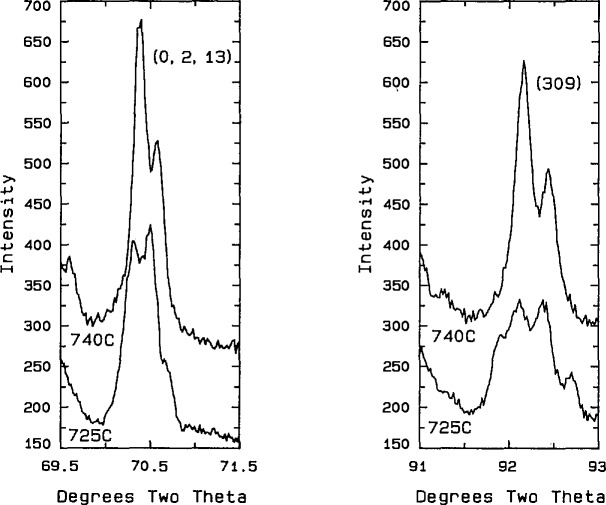
x-ray powder diffraction pattern of CaO:1/2Bi_2_O_3_ 1:6 quenched from 740 °C (rhombohedral indexing) and 725 °C (orthorhombic indexing).

**Flg. 13:**
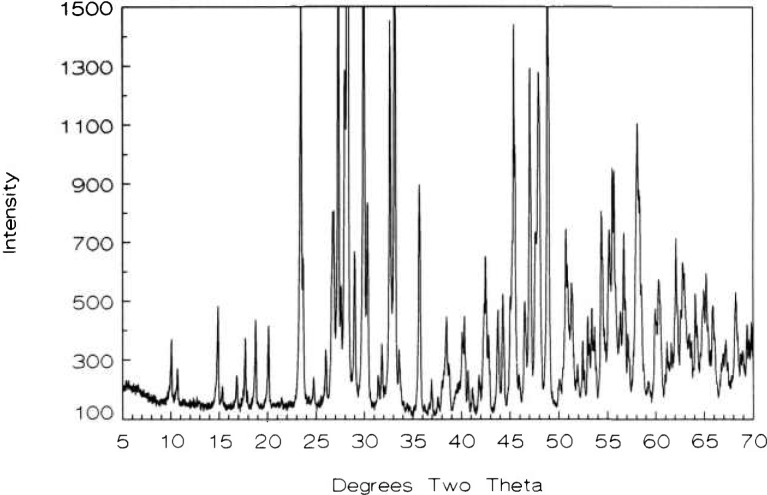
X-ray powder diffraction pattern of the CaBi_2_O_4_ compound.

**Table 1a:** Experimental data for the system CaO-Bi_2_O_3_-CuO

Spec. no.		Composition mole percent			Heat treatment^b^ temp °C		Phys. obser.^c^	Results of x-ray diffraction^d^
	CaO	1/2Bi_2_O_3_	CuO	Initial	final	Time h		
	100	0	0					
				500				CaCO_3_
				600				CaO+CaCO_3_
				600×2				CaO
	66.7	0	33.3					
				700				
				850				
				1000×3				C_2_Cu
	60	0	40					
		*nitrates*		500				CaO+CuO
					750	48		CaO+CuO+C_1−_*_x_*Cu
					$\begin{array}{c} 700\\ 800 \end{array}\}$	$\begin{array}{c} 24\\ 12 \end{array}\}$		CuO+CaO+C_2_Cu
				750×2				
					900			C_2_Cu+CuO
					745	2.0−O_2_		C_2_Cu+CuO
					$\begin{array}{l} 800\\ 875\times 2 \end{array}\}$			C_2_Cu+CuO
					950			C_2_Cu+CuO
					980	16		C_2_Cu+CuO
					990	0.66		C_2_Cu+CuO+CCu_2_
					990	14.0		C_2_Cu+CCu_2_
					1000			C_2_Cu+CCu_2_
					1000×2			C_2_Cu+CCu_2_
					10000×3			C_2_Cu+CCu_2_
					1007	0.16		
					1011	1.0		C_2_Cu+Cu_2_O+CCu_2_
					1014	0.5		C_2_Cu+Cu_2_O
	50	0	50					
#1		*ppt. hydrox-carb.*		450				
					740	6.0		C_1_*_−x_*Cu+CaO+CuO_tr_
					740	15.0		C_1_*_−x,_* Cu+CaO
				740				C_1_*_−x_*Cu+CaO
					800	16.0		C_2_Cu + CuO
#2		*ppt. hydrox-carb.*		500				CuO+C_1_*_−x_*Cu
					550			CuO+C_1_*_−x_*Cu + CaO
					600			C_1_*_−x_*Cu+CaO+ CuO
					650			C_1_*_−x_*Cu+CuO+CaO
					700			C_1_*_−x_*Cu+CuO+Ca(OH)_2_
					740			C_1_*_−x_*Cu+CaO+CuO
					740	62.5		C_1_*_−x_*Cu+CaO+C_2_Cu_tr_
					760			C_1_*_−x_*Cu+CaO+CuO
					780			C_1_*_−x_*Cu+CaO+CuO
					800			C_1_*_−x_*Cu+CaO+CuO_tr_
#3				600				CuO+CaO+CaCO_3_+C_1_*_−x_*Cu
				600×2				CuO+CaO+CaCO_3_+C_1_*_−x_*Cu
				600×3				CuO+CaO+CaCO_3_+C_1_*_−x_*Cu
				600×4				CuO+CaO+CaCO_3_+C_1_*_−x_*Cu
				675				CuO+CaO+C_1_*_−x_*Cu
				675×5				C_1_*_−x_*Cu+CaO+CuO
				675×11				C_1_*_−x_*Cu+CaO+CuO
				675×16				C_1_*_−x_*Cu+CaO+CuO
				675×21				C_1_*_−x_*Cu+CaO+CuO
				675×26				C_1_*_−x_*Cu+CaO+CuO
				675×31				C_1_*_−x_*Cu+CaO+CuO
				675×36				C_1_*_−x_*Cu+CaO+CuO
				750×2				CaO+CuO+C_2_Cu
				850				CaO+CuO+C_2_Cu
				900				C_2_Cu+CuO+CaO
				600				
				750				
				900				C_2_Cu+CuO+CaO
					675	70		C_2_Cu+CuO+CaO
					675×4			C_2_Cu+CuO+CaO
#4		*nitrates*		500				
				600				
					995	1.0		C_2_Cu+CuO+CCu_2_
					1007	10.0		C_2_Cu+CCu_2_+Cu_2_O
					1011	1.0		C_2_Cu+Cu_2_O+CCu_2_
					1013	1.0		C_2_Cu+Cu_2_O+CCu_2tr_
					1007 1013	$\begin{array}{l} 10\\ 24 \end{array}\}$		C_2_Cu+Cu_2_O+CuO+CCu2_tr_
					1014	0.5		C_2_Cu+Cu_2_O+CuO+CCu_2tr_
					1018	0.5		C_2_Cu+Cu_2_O+CuO+CCu_2tr_
					1022	0.5	n.m.	C_2_Cu+Cu_2_O+CuO+CCu_2tr_
					1028	0.5	p.m.	C_2_Cu+Cu_2_O+CCu_2tr_
					1032	0.5	p.m.	C_2_Cu+Cu_2_O+CCu_2tr_
					1036	0.5	p.m.	CaO+C_2_Cu+Cu_2_O+CCu_2tr_
					1040	0.5	p.m.	CaO+C_2_Cu+Cu_2_O+CCu_2tr_
#5		*citrates*			700	22		C_1_*_−x_*Cu+CaO
					700	18−O_2_		C_1_*_−x_*Cu+CaO
	47.37	0	52.63					
		(9:10)						
		*citrates*			700	18		C_1_*_−x_*Cu+CaO
					700	78−O_2_		C_1_*_−x_*Cu+CaO
	45.45	0	54.54					
		(5:6)						
		*citrates*			700	18		C_1_*_−x_*Cu
					700	21−O_2_		C_1_*_−x_*Cu+CaO
					700	39−O_2_		C_1_*_−x_*Cu+CaO
					700	78−O_2_		C_1_*_−x_*Cu+CaO
	45.33	0	54.67					
		*citrates*			700	86−O_2_		C_1_*_−x_*Cu
	45.20	0	54.80					
		*citrates*			700	16		
					700	24−O_2_		C_1_*_−x_*Cu
	44.95	0	55.05					
		*citrates*		700	16			
					700	24−O_2_		C_1_*_−x_*Cu+CuO_tr_
	44.70	0	55.30					
		*titrates*		700	16			
					700	24−O_2_		C_1_*_−x_*xCu+CuO
	40	0	60					
		*citrates*		700	60			C_1_*_−x_*Cu+CuO
				700	18−O_2_			C_1_*_−x_*Cu+CuO
				800				
	33.3	0	66.7					
				800				
				875×2				C_2_Cu+CuO
					965	25.0		C_2_Cu+CuO
					1000	19.0		CCu_2_+C_2_Cu+CuO
					1000×2			CCu_2_+C_2_Cu+CuO
	30	0	70					
#1		*nitrates*		500				CuO+CaO
				750				CuO+CaO
				770				CuO+CaO
				750×2				CuO+CaO+C_2_Cu
				990				CuO+C_2_Cu
				500				
					980	16.0		CuO+C_2_Cu
					990	22.0		CCu_2_+CuO+C_2_Cu
					1000	16.0		CCu_2_+Cu_2_O_tr_+C_2_Cu_tr_
					1010	0.5		CCu_2_+Cu_2_O+C_2_Cu_tr_
					1014	0.5		Cu_2_O+C_2_Cu+CCu_2_
					1016	24.0		Cu_2_O+C_2_Cu+CCu_2tr_
#2		*citrates*			700	86−O_2_		
	25	0	75					
#1				600				
				750				
					950			CuO+C_2_Cu
					975			CuO+C_2_Cu
					1000			CCu_2_+CuO+Cu_2_O+C_2_Cu
					1025			Cu_2_O+C_2_Cu+CuO
#2		*nitrates*			450			
				500				
				600				CuO+CaO
					750	72−O_2_		CuO+C_1_*_−x_*Cu
					770	48−O_2_		CuO+C_1_*_−x_*Cu
					780	68−O_2_		CuO+C_1_*_−x_*Cu
					790	30−O_2_		CuO+C_1_*_−x_*Cu+CaO_tr_
					800	36−O_2_		CuO+C_1_*_−x_*Cu
					820	42−O_2_		CuO+C_1_*_−x_*Cu
					830	72−O_2_		CuO+C_1_*_−x_*Cu+C_2_Cu_tr_
					840	36-O_2_		CuO+C_1_*_−x_*Cu+C_2_Cu
					880	36−O_2_		CuO+C_2_Cu
					750	54		CuO+CaO+C_1_*_−x_*Cu
					760	120		CuO+C_2_Cu
					780	120		CuO+C_2_Cu
					800	20		CuO+C_2_Cu+CaO
					840	64		CuO+C_2_Cu
					1012	1.0	p.m.	Cu_2_O+C_2_Cu+CCu_2_
					1020	0.5	p.m.	Cu_2_O+C_2_Cu+CCu_2_+CaO
	20	0	80					
		*nitrates*		500				
				600				
					1007	1.0		CuO+CCu_2_+Cu_2_O_tr_
					1011	1.0		CCu_2_+Cu_2_O+CuO
					1014	0.16	p.m.	CCu_2_+Cu_2_O+CuO
					1016	0.5	p.m.	Cu_2_O+C_2_Cu+CuO+CCu_2_
					1020	0.5	c.m.	Cu_2_O+C_2_Cu+CuO+CCu_2_
	15	0	85					
		*nitrates*		500				
				600	1016	0.16	p.m.	CuO+Cu_2_O+CCu_2_
					1020	0.33	c.m.	Cu_2_O+CuO+CCu_2_
	10	0	90					
		*nitrates*		500				
				600	1020	0.16	p.m.	Cu_2_O+CCu_2_+CuO
	5	0	95					
		*nitrates*		500				
				600	1016	0.16	p.m.	CuO+Cu_2_O+CCu_2_
					1020	0.16	p.m.	CuO+Cu_2_O+CCu_2_
	10	90	0					
				700				
				750				rhomb+fcc′
					850	0.33	s.m.	rhomb+fcc′+fcc″
					860	0.33	p.m.	
					870	0.33	c.m.	fcc′+rhomb_tr_
	20	80	0					
				700				
				750				rhomb
					650			rhomb
					835	0.33		rhomb+fcc′
					875	0.33	s.m.	rhomb+fcc′
					875	0.66	s.m.	rhomb+fee′
					890	0.33	c.m.	rhomb+fcc′
					$\begin{array}{l} 700\to 875\\ 875\to 650 \end{array}\}$	at 3°/h		rhomb+C_5_B_14_
					$\begin{array}{l} 750\to 870\\ 870\to 845 \end{array}\}$	at 1°/h		rhomb
	23	77	0					
				700				rhomb+C_2_B_3_
				800				rhomb+C_2_B_3_
					840	0.5	fcc′	
					870	0.33	n.m.	fcc′
					880	0.33	n.m.	fcc′+rhomb
					880	0.33	n.m.	fcc′
					890	0.33	cm.	fcc′
				850				
				750×2				rhomb
	25	75	0					
				700				
				750				rhomb+CB_2_+C_5_B_14_
					650	16		rhomb+C_5_B_14_
					750	1		rhomb
					780	0.5		rhomb
					800	1		rhomb
					950	1.2	c.m	fcc′
				850				rhomb
				750×2				rhomb
	26	74	0					
				700				
				750				rhomb+C_2_B_3_
					820	0.33	n.m.	fcc′+rhomb_tr_
					880	0.33	p.m.	fcc′+bcc_tr_
					890	0.33	cm.	fcc′
	26.32	73.68 (5:14)	0					
#1				750				
				650				C_5_B_14_+rhomb+C_2_B_3_
					750	16		rhomb+C_2_B_3_+C_5_B_14_
					1000	1.75	c.m.	fcc′+bcc_tr_
					650			C_5_B_14_
#2				650×2				rhomb+C_2_B_3_+CB_2_
				650×5				rhomb+CB_2_+C_5_B_14_
				750×3				rhomb+C_5_B_14_+CB_2tr_
#3				750				rhomb+C_2_B_3_
				750×2				
				925		0.33	c.m.	fcc′
				750×3				rhomb+CB_2_+C_5_B_14_
					925	0.33	c.m.	fcc′
					1000	1.0	c.m.	
					650	16		C_5_B_14_
					650	336		C_5_B_14_
				750×5				rhomb+CB_2_+C_5_B_14_
					700	100 MPa		rhomb
	27.27	72.72 (3:8)	0					
				750				
					650			rhomb+CB_2_+C_2_B_3_+C_5_B_14_
				750×5				CB_2_+C_5_B_14_+rhomb
					750	16.0		CB_2_+rhomb+ C_5_B_14_
				850				
				750×2				C_5_B_14_+CB_2_+rhomb_tr_
	28	72	0					
				700				
				750				
					860	0.33		fcc′
					870	0.33	n.m.	fcc′
					880	0.33	p.m.	fcc′
					900	0.66	c.m.	fcc′
	30	70	0					
				750				
					650			CB_2_+C_5_B_14_+C_2_B_3_+rhomb
				750×5				CB_2_+C_5_B_14_+rhomb
					750	1.33		CB_2_+C_5_B_14_+rhomb
				850				
				750×2				CB_2_+C_5_B_14_+rhomb
	33.33 (1:2)	66.67	0					
#1				800				
				1000	0.166		c.m.	
				750				C_2_B_3_+C_5_B_14_+CB_2_
					750	16.0		C_2_B_3_+C_5_B_14_+rhomb
#2				700				
				750				CB_2_+C_5_B_14_+C_2_B_3_+rhomb
					65	96		CB_2_+C_5_B_14_+C_2_B_3_
					850	16		fcc′+bcc_tr_
				800				fcc′+C_2_B_3_
				850				
				750×2				CB_2_+rhomb+C_5_B_14_
				1000		1.75	c.m.	fcc′+bcc_tr_
				650		16		CB_2_+C_2_B_3tr_
#3				750×5				CB_2_+rhomb+C_2_B_3tr_
					750	1.33		CB_2_+rhomb
					$\begin{array}{l} 925\\ 700 \end{array}\}$	0.13 312	c.m.	CB2+C2B3†
					$\begin{array}{l} 1000\\ 650 \end{array}\}$	1.0 17	c.m.	CB_2_+C_5_B_14_+C_2_B_3_
				650×4				
				650×5				CB_2_+C_2_B3_tr_+C_5_B_14tr_
				700				CB_2_+C_5_B_14_+C_2_B_3tr_
				750×3				CB_2_
				750				C_2_B_3_+rhomb
				750×3				CB_2_+C_2_B_3tr_
				750×5				CB_2_+C_2_B_3tr_
					650 100 MPa			CB_2_+C_2_B_3_
#5	*lactate*			450				
				650×3				CB_2_+C_5_B_14_
				650×4				CB_2_+C_5_B_14_
					700			CB_2_+C_5_B_14_
					750			CB_2_+C_2_B_3_+rhomb+C_5_B_14tr_
	35	65	0	750				C_2_B_3_+rhomb+fcc
					770	60		C_2_B_3_+rhomb+fcc′
						780 0.33		C_2_B_3_+rhomb+fcc
					790	0.66		C_2_B_3_+fcc′+rhomb_tr_
					820	0.33		C_2_B_3_+fcc′
					830	0.33		C_2_B_3_+fcc′
					830	8.0		C_2_B_3_+fcc′+bcc_tr_
					840	0.33		C_2_B_3_+fcc′+bcc_tr_
					840	13.0		bcc
					850	0.33		bcc+fcc′+unknown
					850	1.0		bcc
					920	0.16	p.m.	bcc+fcc′
	37.5	62.5 (3:5)	0					
				750				
				650				C_2_B_3_+CB_2_+C_5_B_14_
				750×5				C_2_B_3_+CB_2_
	40	60 (2:3)	0					
#1				750				
				650				C_2_B_3_+CB_2_+C_5_B_14_+CB+CaO
				750×5				C_2_B_3_
#2				750				
				650				
				750×5				C_2_B_3_
				800				C_2_B_3_
				850				C_2_B_3_
					900	1.0		bcc
#3				700				
				700×5				C_2_B_3_
				850				C_2_B_3_
				900				bcc+C-mon+unknown
					750			C_2_B_3_
#4				700				
				800				
				900×2				C_2_B_3_+unknown
				750				C_2_B_3_
					700	240		C_2_B_3_
					875	16		bcc
					$\begin{array}{l} 1000\\ 700 \end{array}\}$	1.0 240		C_2_B_3_
#5				700				
				850				
				900×2				
				825				C_2_B_3_
#6				700				
				750				
					860	0.33		bcc
					935	0.33	n.m.	bcc
					950	0.33	p.m.	bcc
	41.18	58.82 (7:10)	0					
				750				
				650				C_2_B_3_+CB_2_+CB+CaO
					825	17		C_2_B_3_+CB
					900	20		bcc+C-mon+fcc′
				750×5				C_2_B_3_+CB
	42.86	57.14 (3:4)	0					
				700				
				750				
				850				C_2_B_3_+CB
	45	55	0					
				700				
				750				C_2_B_3_+CB+CB_2_+CaO
					650	96		C_2_B_3_+CB+CaO
					850	16		C_2_B_3_+CB+CaO
					870	0.66		bcc+CB
					890	0.33		bcc+C-mon+CB
					900	0.33		bcc+C-mon+CB_tr_
					900	1.00		bcc+C-mon
					940	1.00		bcc+CB+C-mon
					880	1.00		
					950	0.33	p.m.	bcc+C-mon_tr_
					1000	1.75	c.m.	bcc
	48	52	0					
				700				
				800				
				900				CB+bcc
					955	0.33		C-mon+bcc+CaO
					960	0.33		bcc+C-mon+CaO
					940	0.33		
					970	0.33	p.m.	bcc+CaO
	50	50	0					
#1				700				
				750				CB+C_2_B_3_+CaO
					650	96		CB+C_2_B_3_+CaO
					850	16		CB+C_2_B_3_
					900	1.0		CB
				900				
					940	1.0		
					$\begin{array}{l} 940\\ 820 \end{array}\}$	2.0 15		C-mon CB+Czmon
					1000	1.0	c.m.	bcc+CaO
#2				750				C_2_B_3_+CB+CaO
					860	10.0		CB
					880	1.0		CB+unknown+CaO
					940	0.33		C-mon
					940	2.0		C-mon
					950	0.25		C-mon
					960	0.5	n.m.	C-mon+bcc+CaO
					970	0.33	p.m.	bcc+CaO
				940		2.0		C-mon
					850	3.0		C-mon
					880	11.0		CB
					$\begin{array}{l} 1000\\ 940 \end{array}\}$	0.16 24.0		bcc+CaO
#3				700				
				800				
				900				CB
				825				CB
					940			CB
				940				CB
#4				700				
				750×4				
				850				CB
				900				CB
	53.85	46.15 (7:6)	0					
#1				750				
				650				C_2_B_3_+rhomb+CB+CaO
				750×5				CB+CaO
#2				750				
				650				
				900				CB+CaO
#3				700				
				800				
				900				CB+CaO
				825				CB+CaO
					940	16.0		CB+CaO
	54	46	0					
				750				
				650				
					930	2.0	n.m.	C-Mon+CaO
					940	2.0		
					920	2.0		C-Mon+CaO
	57.14	42.86 (4:3)	0					
				750				
				850				CB+CaO+C_2_B_3tr_
				900				CB+CaO
	60	40	0					
#1				900				CB+CaO
				900×2				CB+CaO
#2				750				
				650				CB+C_2_B_3_+CaO
				750×5				CB+CaO
	66.67	33.33	0					
				750×2				CB+CaO
					920	0.33	n.m.	CB+CaO
					930	0.33	n.m.	C-Mon+CaO
					940	0.33	n.m.	C-Mon+CaO
					950	0.33	n.m.	C-Mon+CaO
					960	0.33	n.m.	bcc+CaO
	71.43	28.57 (5:2)	0					
				750×5				CB+CaO
	11.11	44.44	44.44					
				700				
				750				rhomb+CuO+B_2_Cu
				750×5				rhomb+CuO+B_2_Cu
	20	40	40					
				700				
				750				CuO+rhomb+CB_2_
				750×5				CuO+CB_2_+rhomb
	33.33	33.33	33.33					
				700				
				750				CB+C_2_B_3_+CuO
				750×5				CB+C_2_B_3_+CuO
	44.02	7.14	48.84					
	*Ca_4_Bi_6_O_13_+Ca_2_Bi_2_O_5_+C_1−x_CuO_2_*							
		1:1:10						C_1−_*_x_*Cu+C_2_B_3_+CB
				700				C_1−_*_x_*Cu+CB+C_2_B_3_
				700×2				C_1−_*_x_*Cu+CB+C_2_B_3tr_
				700×3				C_1−_*_x_*Cu+CB
				700×4				C_1−_*_x_*Cu+CB
	44.44	22.22	33.33					
	*Ca_2_CuO_3_+Bi_2_CuO_4_*							
		2:1						C_2_Cu+B_2_Cu
				700				C_2_Cu+C_2_B_3_+B_2_Cu+CuO
				700×2				C_2_Cu+C_2_B_3_+B_2_Cu+CuO+CB
				700×3				C_2_Cu+C_2_B_3_+CuO+CB+B_2_Cu^e^
	45	45	10					
				700				
				750				
					920	0.33	p.m.	bcc+C-mon+CaO
					940	0.33	c.m.	bcc+CaO+C-mon_tr_
	49	49	2					
				700				
				750				
					900	0.33		CB
					910	0.33		CB
					915	16.0	p.m.	CB
					930	0.33	p.m.	bcc+CaO
	50	25	25					
				700				
				750				CB+CuO+CaO
					800			CB+CuO+CaO
				750×5				CB+CuO+CaO
	54	23	23					
				700				
				750×6				CB+CaO+CuO+C_1−_*_x_*Cu
	54.95	14.63	30.41					
	*Ca_4_Bi_6_O_13_+Ca_2_CuO_3_+C_1−x_CuO_2_*							
		1:7:3						C_2_B_3_+C_2_Cu+C_1−_*_x_*Cu
				700				C_2_B_3_+C_2_Cu+C_1−_*_x_*Cu+CB
				700×2				C_2_Cu+C_1−_*_x_*Cu+CB+C_2_B_3_
				700×3				C_2_Cu+C_1−_*_x_*Cu+CB+C_2_B_3_
	56	24	20					
	*Ca_4_Bi_6_O_13_+Ca_2_CuO_3_*							
		1:5						C_2_Cu+C_2_B_3_
#1				700				C_2_Cu+C_2_B_3_+CB
				700×2				C_2_Cu+C_2_B_3_+CB+CuO
				700×3				C_2_Cu+CB+C_2_B_3_+CuO
					700	O_2_		C_2_Cu+CB+C_2_B_3_+CuO
					750			C_2_Cu+CB+C_2_B_3_+CuO_tr_
#2					750×2			C_2_Cu+CB+C_2_B_3_+CuO_tr_
					750×2			
					700	336		C_2_Cu+CB+CuO_tr_
#3	+C_1−_*_x_*Cu_tr_							CB+C_2_Cu+C_2_B_3_
#4	+C_1−_*_x_*Cu(more)							CB+C_2_Cu+C_2_B_3_+C_1−_*_x_*Cu
					700			CB+C_2_Cu+C_2_B_3_+C_1−_*_x_*Cu
					700×2			
					700×3^f^			
					700×4^f^			
					700×5^f^			
	57.14	9.52	33.33					
	*Ca_2_CuO_3_+Bi_2_CuO_4_*							
	6:1							C_2_Cu+B_2_Cu
				700				C_2_Cu+B_2_Cu+C_2_B_3_+CuO
				700×2				C_2_Cu+C_2_B_3_+B_2_Cu+CuO
				700×3				C_2_Cu+CB+C_2_B_3_+CuO
	60	20	20					
				700				
				750				CB+CaO+CuO
				750×5				CB+CaO+Ca_1−_*_x_*Cu+CuO
				750×9				CB+CaO+Ca_1−_*_x_*Cu+CuO
	61.29	19.35	19.35					
	*Ca_2_CuO_3_+Ca_7_B_6_O_16_*							
		6:1		750×2				CB+C_2_Cu+CaO
					700	336		CB+C_2_Cu+CaO_tr_
	70	15	15					
				700				
				750×5				CaO+CB+Ca_1−_*_x_*Cu+CuO
					800			CaO+CB+Ca_1−_*_x_*Cu+CuO
					850			CaO+CB+C_2_Cu
					900			CaO+C-mon+C_2_Cu
					900			
					750			CaO+CB+C_2_Cu
					$\begin{array}{l} 900\\ 750 \end{array}\}$			CaO+Czmon+C_2_Cu
					900×7,126			

a Starting materials CaCO_3_, Bi_2_O_3_, CuO except when listed in italics. Compositions given in italics were formulated from the listed pre-reacted compounds or compositions.

b Specimens were given all previous heat treatments listed in the initial column, sequentially, and held at temperature 16–24 h, with grinding in between, for the number of times shown and then reheated at the final temperature for the indicated number of hours. (if hours are not specified heat treatment was overnight). O_2_=heat treatment in one atmosphere of purified oxygen.

c p.m. = partially melted, c.m. = completely melted, n.m. = no melting, s.m. = slightly melted.

d Compounds are listed in order of estimated amounts, most prevalent first.

tr=trace, just barely discernable.

C_2_Cu = Ca_2_CuO_3_

C_1−_*_x_*Cu = Ca_1−_*_x_*CuO_2_

CCu_2_=CaCu_2_O_3_

rhomb=rhombohedral solid solution

fcc=face centered cubic solid solution; symmetry often distorted and generally with superstructure

fcc′-very slight rhombohedral distortion of cubic symmetry, with incommensurate superstructure perpendicular to the hexagonal c* (corresponding to α′, of [20].

fcc″-metastablephase with larger rhombohedral distortion of cubic symmetry, with superstructure equal to 42 and faint incommensurate superstructure perpendicular to the hexagonal [*hOl*] plane.

bcc=body centered cubic solid solution; symmetry often distorted and generally with superstructure.

C_5_B_14_ = Ca_5_Bi_14_O_26_

CB_2_=CaBi_2_O_4_

C_2_B_3_ = Ca_4_Bi_6_O_13_

CB = Ca_2_Bi_2_O_5_(triclimic)

C-mon = metastable C-centered monochnic phase near Ca_6_Bi_7_O_16.5_.

e Although Ca_4_Bi_6_O_13_ has formed during first 700 °C heat treatment, further heating and grinding resulted in formation of Ca_2_Bi_3_O_5_, which increased with the third heat treatment, indicating that the 2:3 phase was formed metastably but the 1:1 compound is the stable phase.

f Amount of 2:3 decreasing and amount of Ca_1_*_−x_*CuO_2_ may be increasing very slightly.

**Table 1(b):** Experimental conditions for crystal growth experiments

Charge	Flux	Container	Temperature cycle	Results
CaO:1/2Bi_2_O_3_ 1:6	(KNa)Cl	Small dia Au sealed	700 °C 595 h	biaxial xtals Rhomb (Orth)
90 wt%	10 wt%			

CaO:1/2Bi_2_O_3_ 1:4		Small dia Au sealed	700→875 °C @ 10 °C/h 875→650 °C @ 3 °C/h	

CaO:1/2Bi_2_O_3_ 5:14	(KNa)Cl	Large dia Pt sealed	750 °C→645 °C @ 1 °C/h 645 °C 64 h	
20 wt%	80 wt%			
CaO:1/2Bi_2_O_3_ 5:14	(KNa)Cl	large dia Pt	750 °C→645 °C @ 1 °C/h	
20 wt%	80 wt%		645 °C 64 h	
CaO:1/2Bi_2_O_3_ 5:14	10μLH_2_O	Small dia Au sealed	Hydrothermal unit 700 °C 100 MPa	
CaO:1/2Bi_2_O_3_ 5:14	(KNa)Cl	Large dia Au sealed	650 °C→750 °C @ 10 °C/h	
80 wt%	20 wt%		750 °C→640 @ 1 °C/h	
CaO:1/2Bi_2_O_3_ 5:14	None	Small dia Au open	900 °C, 20 min. quenched (liq N_2_ cooled He cup) crushed	
		Small dia Au open	780 °C 67.5 h quenched (liq N_2_ cooled He cup)	fcc′
CaO:1/2B_2_O_3_ 5:14	None	Small dia Au sealed	925 °C→850 °C @ 3 °C/h 850 °C 24 h quenched (liq N_2_ cooled He cup)	Ca_5_Bi_14_O_26_
		Small dia Au open	650 °C 2 weeks	
CaO:1/2Bi_2_O_3_ 5:14	None	Small dia Au sealed	925 °C→850 °C @ 3 °C/h	
		Small dia Au open	650 °C 16 h	

CaO:1/2Bi_2_O_3_ 3:8	None	Small dia Au open	900 °C 22 h quenched (liq N_2_ cooled He cup) crushed	
		Small dia Au open	−800 °C 3 d quenched (liq N_2_ cooled He cup)	
			−760 °C 15 min pulled from furnace	
			−800 °C 1 h quenched (liq N_2_ cooled He cup)	
			−760 °C 10 min quenched (liq N_2_ cooled He cup)	fcc″
CaO:1/2Bi_2_O_3_ 33:67	(KNa)Cl	Small dia Au sealed	775 °C (18h)→645 °C @ 1 °C/h	
80 wt%	20 wt%			
CaO:1/2Bi_2_O_3_ 33:67	(KNa)Cl	Small dia Au sealed	775 °C(18h)→645 °C @ 1 °C/h	CaBi_2_O_4_
20 wt%	80 wt%			
CaO:1/2Bi_2_O_3_ 33:67	(KNa)Cl	Small dia Au sealed	775 °C(18h)→645 °C @ 1 °C/h	CaBi_2_O_4_
50 wt%	50 wt%			

CaO:1/2Bi_2_O_3_ 1:2	(KNa)Cl	Large dia Ft sealed	750 °C→645 °C @ 1 °C/h 645 °C 64 h	
20 wt%	80 wt%			
CaO:1/2Bi_2_O_3_ 1:2	(KNa)Cl	Large dia Pt	750 °C→645 °C @ 1 °C/h 645 °C 64 h	
20 wt%	80 wt%			
CaO:1/2Bi_2_O_3_ 1:2	(KNa)Cl	vycor cruc.	675 °C 144 h	
20 wt%	80 wt%			
CaO:1/2Bi_2_O_3_ 1:2	None	Small dia Au sealed	925 °C→850 °C @ 3 °C/h 850 °C 24 h quenched (liq N_2_ cooled He cup) crushed	
		Small dia Au open	500 °C→700 °C @ 3 °C/h 700 °C 168 h	
CaO:1/2Bi_2_O_3_ 1:2	None	Small dia Au sealed	925 °C→850 °C @ 3 °C/h	
		Small dia Au open	650 °C 16 h	
CaO:1/2Bi_2_O_3_ 1:2	(KNa)Cl	Large dia Au sealed	650 °C→750 °C @ 10 °C/h	
80 wt%	20 wt%		750 °C→640 °C @ 1 °C/h	
CaO:1/2Bi_2_O_3_ 1:2	10μL H_2_O	Small dia Au sealed	Hydrothcrnal unit 700 °C 100 MPa	
CaO:1/2Bi_2_O_3_ 1:2	None	Large dia Au sealed	750 °C→875 °C @ 25 °C/h 875 °C→845 °C @ 1 °C/h	
CaO:1/2Bi_2_O_3_ 1:2	None	Small dia Au sealed	925 °C 10 min quenched (liq N_2_ cooled He cup) crushed to a fine powder	
		Small dia Au open	500 °C→700 °C @ 3 °C/h	
				
CaO:1/2Bi_2_O_3_ 2:3	None	Small dia Au sealed	1000 °C→900 °C @ 1°C/h crushed	
		Small dia Au sealed	825 °C 190 h furnace cooled	
CaO:1/2Bi_2_O_3_ 2:3	None	Small dia Au sealed	1000 °C 1 h quenched (liq N_2_ cooled He cup)	Ca_4_Bi_6_O_13_
			875 °C 260 h	
CaO:1/2Bi_2_O_3_ 2:3	(KNa)Cl	Large dia Au sealed	840 °C→640 °C @ 1 °C/h	
98 wt%	2 wt%			
CaO:1/2Bi_2_O_3_ 2:3	(KNa)Cl	Large dia Au sealed	840 °C→640 °C @ 1 °C/h	
80 wt%	20 wt%			
CaO:1/2Bi_2_O_3_ 2:3	(KNa)Cl	Large dia Au sealed	840 °C→640 °C @ 1 °C/h	
50 wt%	50 wt%			
CaO:1/2Bi_2_O_3_ 2:3	(KNa)Cl	Large dia Au sealed	840 °C→640 °C @ 1 °C/h	
20 w%	80 wt%			

CaO:1/2Bi_2_O_3_ 7:10	(KNa)Cl	Large dia Pt sealed	750 °C→645 °C @ 1 °C/h 645 °C 64 h	Ca_4_Bi_6_O_13_
20 wt%	80 wt%			
CaO:1/2Bi_2_O_3_ 7:10	(KNa)Cl	Large dia Pt	750 °C→645 °C @ 1 °C/h	
20 wt%	80 wt%			

CaO:1/2Bi_2_O_3_ 6:7	CaCl_2_	Large dia Au open	900 °C 20 h	
80 wt%	20 wt%			

CaO:1/2Bi_2_O_3_ 1:1	(KNa)Cl	Small dia Au sealed	650 °C→950 °C @ 100 °C/h 950 °C→900 °C @ 1 °C/h	
80 wt%	20 wt%			
CaO:1/2Bi_2_O_3_ 1:1	(KNa)Cl	Small dia Au sealed	650 °C→950 °C @ 100 °C/h 950 °C→900 °C @ 1 °C/h	
50 wt%	50 wt%			
CaO:1/2Bi_2_O_3_ 1:1	(KNa)Cl	Small dia Au sealed	650 °C→950 °C @ 100 °C/h 950 °C→900 °C @ 1 °C/h	
20 wt%	80 wt%			

CaO:1/2Bi_2_O_3_ 7:6	(KNa)Cl	Large dia Pt sealed	750 °C→645 °C@ 1 °C/h 645 °C 64 h	Ca_2_Bi_2_O_5_
20 wt%	80 wt%			
CaO:1/2Bi_2_O_3_ 7:6	(KNa)Cl	Large dia Pt	750 °C→645 °C @ 1 °C/h	
20 wt%	80 wt%			
CaO:1/2Bi_2_O_3_ 7:6	(KNa)Cl	Large dia Au sealed	900 °C 19.5 h	
80 wt%	20 wt%			

**Table 2:** Crystal structure data

Chemical formula	Symmetry phase (*T* °C)	*a* (Å)	*b* (Å)	c (Å)	*α* degrees	*β* degrees	*γ* degrees
Ca_1−_*_x_*,CuO_2_ *x* =0.172	Fmmm^a^ *T* ~700 °C	2.8047^b^ (7)	6.321 (2)	10.573 (2)			
CaO:1/2Bi_2_O_3_ 1:6	$R\bar{3}$ *T* ~750 °C	3.9448 (8)		27.8400 (8)			
	Cmmm *T* ⩽735 °C	6.8188 (3)	3.9531 (2)	27.830 (1)			
CaO:1/2Bi_2_O_3_ 3:8	$R\bar{3}$ *α*′ (*T* ~780 °C)	7.7427 (9)		9.465 (1)			
	B2/m *α*″(*T* ~760 °C)	15.5819 (3)	3.8077 (1)	10.8955 (3)		91.829 (2)	
Ca_5_Bi_12_O_41_	$P\bar{1}$	9.934 (1)	15.034 (2)	15.008 (2)	82.65 (1)	85.27 (1)	
CaBi_2_O_4_	C2/c	16.6295 (8)	11.5966 (5)	14.0055 (6)		134.036 (3)	
Ca_4_Bi_6_O_13_	C2mm	17.3795 (5)	5.9419 (2)	7.2306 (2)			
CaO:1/2Bi_2_O_3_ 9:10	“bcc” *T* ~1000 °C	4.2458 (1)					
Ca_2_Bi_2_O_5_	$P\bar{1}$	10.1222 (7)	10.146 (6)	10.4833 (7)	116.912 (5)	107.135 (6)	92.939 (6)
Ca_6+_*_x_*Sr_6_*_−x_*Bi_14_O_33_ *x*→6	C-centered monoclinic	21.295 (4)	4.3863 (8)	12.671 (2)		102.74 (1)	

a Indicates a subcell.

b Numbers in parentheses indicate uncertainties in final digits.

**Table 3:** x-ray powder diffraction data for the compound Ca_1_*_−x_*CuO_2_

*d* obs(Å)	Rel *I* (%)	2*θ* obs	2*θ* calc^a^	*hkl*
5.273	13	16.80	16.76	002
3.1554	21	28.26	28.21	002
3.0994	1	28.78^b^		
2.8914	6	30.90	30.91	1−8δ,1,1−δ*c*
2.8245	3	31.65	31.66	1−8δ,1,1+δ*c*
2.7106	100	33.02	32.99	022
2.6407	22	33.92	33.89	004
2.4887	23	36.06	36.02	111
2.3218	6	38.75	38.77	1−δ*a*,1,3−δ*c*
2.2207	7	40.59	40.60	1−δ*a*,1,3+δ*c*
2.0720	61	43.65	43.62	113
1.7666	4	51.70	51.72	1−δ*a*,3,1−δ*c*
1.7613	6	51.87	51.84	600
1.7571	6	52.00	51.95	1−δ*a*,1,5−δ*c*
1.7527	8	52.14	52.21	1−δ*a*,3,1+δ*c*
1.6840	2	54.44	54.39	1−δ*a*,1,5+δ*c*
1.6632	10	55.18	55.16	131
1.6306	29	56.38	56.36	115
1.6088	2	57.21	57.23	1−δ*a*,3,3−δ*c*
1.5802	12	58.35	58.34	040
1.5397	18	60.04	60.06	026
1.5200	16	60.90	60.90	133
1.4811	1	62.67^b^		
1.4545	1	63.95^b^		
1.4467	1	64.34^b^		
1.4129	1	66.07^b^		
1.4025	6	66.63	66.64	200
1.3702	1	68.41	68.42	1−δ*a*,1,7−δ*c*
1.3565	12	69.20	69.21	044
1.3471	2	69.75^b^		
1.3208	13	71.35	71.33	1−δ*a*,1,7+δ*c*
1.3186	15	71.49	71.55	135
1.3018	5	72.56	72.59	117
1.2819	5	73.87	73.87	220

a Calculated on the basis of an orthorhombic subcell, Fmmm, *a* =2.8047 (7), *b* =6.321 (2), and *c* = 10.573 (2) Å.

b Superstructure probably not accounted for by δ-vectors.

**Table 4:** x-ray powder diffraction data for the high temperature rhombohedral (Sillen phase) indexing of CaO:1/2Bi_2_O_3_ 1:6

*d* obs(Å)	Rel *I*(%)	2 *θ* obs	2 *θ* calc^a^	*hkl*
9.254	4	9.55	9.52	003
4.633	8	19.14	19.11	006
3.3897	23	26.27	26.26	101
3.3166	31	26.86	26.85	012
3.0922	93	28.85	28.84	009
3.0651	100	29.11	29.09	104
2.9099	56	30.70	30.68	015
2.5896	16	34.61	34.58	107
2.4372	17	36.85	36.84	018
	2	39.90^b^		
2.1578	10	41.83	41.82	1,0,10
	2	43.67^b^		
2.0326	17	44.54	44.52	0,1,11
1.9726	57	45.97	45.98	110
1.9283	1	47.09	47.07	113
1.8554	12	49.06	49.04	0,0,15
1.8149	57	50.23	50.22	116
			50.24	1,0,13
1.7188	24	53.25	53.26	0,1,14
1.7043	8	53.74	53.72	021
1.6953	10	54.05	54.05	202
1.6629	72	55.19	55.19	119
1.6333	16	56.28	56.29	205
1.5694	6	58.79	58.79	027
1.5500	10	59.60	59.58	1,0,16
1.5467	18	59.74	59.74	0,0,18
1.5334	6	60.31	60.31	208
1.4770	12	62.87	62.88	0,1,17
1.4561	2	63.88	63.89	0,2,10
1.4157	6	65.93	65.92	2,0,11
1.3516	7	69.49	69.49	1,1,15
1.3355	12	70.45	70.46	0,2,13
1.2956	8	72.96	72.95	2,0,14
1.2891	10	73.39	73.39	0,1,20
1.2856	11	73.62	73.61	122
1.2693	15	74.73	74.71	214
1.2579	13	75.52	75.53	125
1.2280	4	77.70	77.69	217
1.2171	21	78.53	78.53	1,1,18
1.2105	5	79.04	79.03	128
1.1868	14	80.94	80.95	1,0,22
1.1823	9	81.31	81.33	2,0,17
1.1712	2	82.25	82.24	2,1,10
1.1598	3	83.24	83.22	0,0,24
1.1503	5	84.08	84.09	1,2,11
1.1407	8	84.95	84.93	0,1,23
1.1386	12	85.15	85.13	300
1.1122	1	87.67	87.68	0,2,19
1.1059	13	88.30	88.30	306
			88.31	2,1,13
1.0828	7	90.70	90.68	1,2,14
1.0790	2	91.11	91.10	2,0,20
1.0686	10	92.25	92.24	309
1.0587	2	93.37	93.36	1,0,25
1.0368	2	95.97	95.95	2,1,16
1.0309	7	96.70	96.67	0,0,27
1.0217	8	97.86	97.86	0,1,26
1.0169	8	98.49	98.50	0,2,22
1.0141	9	98.86	98.87	1,2,17
0.9999	3	100.77	100.78	1,1,24
0.9876	3	102.52	102.52	2,0,23
0.9863	4	102.70	102.72	220
0.9707	1	105.04	105.05	3,0,15
0.9469	4	108.88	108.87	131
0.9454	4	109.14	109.15	312
0.9394	8	110.16	110.13	229
0.9341	4	111.10	111.11	315
0.9330	3	111.31	111.33	0,2,25
0.9243	2	112.90	112.91	0,1,29
0.9218	3	113.36	113.38	137
0.9171	7	114.27	114.28	3,0,18
0.9141	10	114.84	114.83	318
0.9076	3	116.14	116.15	2,2,12
0.9072	3	116.22	116.22	2,0,26
0.9038	7	116.92	116.93	2,1,22
0.8970	1	118.35	118.36	1,3,10
0.8875	2	120.45	120.47	3,1,11
0.8832	3	121.43	121.45	1,2,23
0.8686	6	124.95	124.97	1,0,31
0.8665	7	125.50	125.49	1,3,13
0.8554	4	128.46	128.46	3,1,14

a Calculated on the basis of a rhombohedral unit cell, $R\bar{3}$, *a* =3.9448(8) and *c* =27.8400(8) Å.

b Apparently due to an unidentified structure.

**Table 5:** x-ray powder diffraction data for the low temperature orthorhombic indexing of CaO:1/2Bi_2_O3 1:6

*d* obs(Å)	Rel *I*(%)	2 *θ* obs	2 *θ* calc^a^	*hkl*
9.283	1	9.52	9.53	003
4.6405	10	19.11	19.12	006
	1	25.15^b^		
3.3922	17	26.25	26.23	111
3.3190	24	26.84	26.82	112
3.0911	100	28.86	28.85	009
3.0703	84	29.06	29.07	114
2.9127	47	30.67	30.66	115
2.5911	14	34.59	34.57	117
2.4391	13	36.82	36.82	118
2.4359	14	36.87	36.88	208
	1	38.29^b^		
	1	38.90^b^		
	1	40.76^b^		
2.1588	7	41.81	41.81	1,1,10
2.1563	7	41.86	41.87	2,0,10
	1	43.01^b^		
2.0339	15	44.51	44.51	1,1,11
2.0326	1	44.54	44.56	2,0,11
1.9775	25	45.85	45.87	020
1.9726	40	45.97	46.00	021
	1	47.17^b^		
	1	48.20^b^		
1.8550	5	49.07	49.06	0,0,15
1.8152	51	50.22	50.24	1,1,13
1.8142	49	50.25	50.27	316
	1	51.92^b^		
	1	52.07^b^		
	2	52.90^b^		
1.7188	22	53.25	53.26	1,1,14
1.7174	15	53.30	53.30	2,0,14
1.7070	6	53.65	53.66	221
1.7011	5	53.85	53.84	401
1.6976	6	53.97	53.98	222
1.6924	5	54.15	54.16	402
1.6660	34	55.08	55.10	029
1.6618	54	55.23	55.23	319
1.6607	47	55.27	55.28	224
1.6563	28	55.43	55.45	404
1.6343	10	56.24	56.23	225
1.6298	9	56.41	56.41	405
	1	56.98^b^		
	2	58.34^b^		
1.5704	4	58.75	58.73	227
1.5662	4	58.92	58.90	407
1.5464	18	59.75	59.76	0,0,18
1.5348	5	60.25	60.26	2,2,18
1.5309	3	60.42	60.43	408
	1	61.15^b^		
1.4764	10	62.90	62.89	1,1,17
1.4753	9	62.95	62.93	2,0,17
1.4567	1	63.85	63.84	2,2,10
	2	63.90^b^		
1.4532	1	64.02	64.00	4,0,10
1.4164	3	65.89	65.87	2,2,11
1.4139	3	66.02	66.03	4,0,11
1.3526	4	69.43	69.42	0,2,15
1.3506	4	69.55	69.54	3,1,15
1.3362	9	70.41	70.41	2,2,13
1.3335	10	70.57	70.57	4,0,13
	1	71.22^b^		
1.2964	6	72.91	72.91	2,2,14
1.2942	6	73.05	73.06	4,0,14
1.2889	9	73.40	73.40	1,1,20
1.2852	7	73.65	73.64	422
1.2719	6	74.55	74.54	134
1.2694	8	74.72	74.73	424
1.2678	9	74.83	74.84	514
1.2603	6	75.35	75.36	135
1.2575	7	75.55	75.55	425
1.2562	7	75.64	75.66	515
	2	77.45^b^		
1.2263	3	77.83	77.83	517
1.2167	17	78.56	78.58	3,1,18
1.1866	13	80.96	80.97	1,1,22
1.1862	12	80.99	81.01	2,0,22
1.1734	2	82.06	82.08	1,3,10
1.1699	1	82.36	82.37	5,1,10
1.1594	2	83.27	83.25	0,0,24
1.1496	3	84.14	84.12	4,2,11
1.1409	6	84.93	84.95	1,1,23
1.1404	7	84.98	84.99	2,0,23
1.1399	7	85.03	85.02	330
1.1364	5	85.35	85.35	600
	2	85.60^b^		
1.1266	1	86.27	86.25	5,1,12
	1	87.50^b^		
1.1075	4	88.14	88.16	1,3,13
1.1070	5	88.19	88.18	336
1.1054	6	88.35	88.34	4,2,13
1.1045	7	88.44	88.45	5,1,13
1.1039	6	88.50	88.51	606
1.0842	3	90.55	90.53	1,3,14
1.0827	4	90.71	90.71	4,2,14
1.0818	4	90.80	90.82	5,1,14
1.0793	3	91.07	91.07	2,2,20
1.0780	3	91.22	91.22	4,0,20
1.0694	5	92.16	92.14	339
1.0666	6	92.47	92.46	609
1.0586	2	93.38	93.39	1,1,25
1.0356	2	96.11	96.10	5,1,16
1.0306	5	96.74	96.72	0,0,27
1.0216	5	97.88	97.89	1,1,26
1.0170	6	98.48	98.48	2,2,22
1.0157	6	98.65	98.62	4,0,22

a Calculated on the basis of an orthorhombic unit cell, Cmmm, *a* = 6.8188(3), *b* =3.9531(2), and *c* =27.830(1) Å.

b Apparently due to an unidentified structure.

**Table 6:** x-ray powder diffraction data for the high temperature rhombohcdral (Sillen phase) indexing versus the orthorhombic indexing of CaO:1/2Bi_2_O_3_ 1:6

2 *θ* obs	Rhombohedral		Orthorhombic
	*hkl*^a^	*hkl^b^*	2 *θ* obs
9.55	003	003	9.52
19.14	006	006	19.11
			25.15^b^
26.27	101	111	26.25
26.86	012	112	26.84
28.85	009	009	28.86
29.11	104	114	29.06
30.70	015	115	30.67
34.61	107	117	34.59
36.85	018	118	36.82
		208	36.87
			38.29^b^
			38.90^b^
39.90^b^			
			40.76^b^
41.83	1,0,10	1,1,10	41.81
		2,0,10	41.86
			43.01^b^
43.67^b^			
44.54	0,1,11	1,1,11	44.51
		2,0,11	44.54
		020	45.85
45.97	110	021	45.97
47.09	113		
			47.17^b^
			48.20^b^
49.06	0,0,15	0,0,15	49.07
50.23	116	1,1,13	50.22
	1,0,13		
		316	50.25
			51.92^b^
			52.07^b^
			52.90^b^
53.25	0,1,14	1,1,14	53.25
		2,0,14	53.30
		221	53.65
53.74	021		
		401	53.85
		222	53.97
54.05	202		
		402	54.15
		029	55.08
55.19	119	319	55.23
		224	55.27
		404	55.43
56.28	205	225	56.24
		405	56.41
			56.98^b^
			58.34^b^
58.79	027	227	58.75
		407	58.92
59.60	1,0,16		
59.74	0,0,18	0,0,18	59.75
		2,2,18	60.25
60.31	208		
		408	60.42
			61.15^b^
62.87	0,1,17	1,1,17	62.90
		2,0,17	62.95
63.88	0,2,10	2,2,10	63.85
			63.90^b^
		4,0,10	64.02
65.93	2,0,11	2,2,11	65.89
		4,0,11	66.02
		0,2,15	69.43
69.49		1,1,15	
		3,1,15	69.55
70.45	0,2,13	2,2,13	70.41
		4,0,13	70.57
			71.22^b^
72.96	2,0,14	2,2,14	72.91
		4,0,14	73.05
73.39	0,1,20	1,1,10	73.40
73.62	112	422	73.65
		134	74.55
74.73	214	424	74.72
		514	74.83
		135	75.35
75.52	125	425	75.55
		515	75.64
			77.45^b^
77.70	217		
		517	77.83
78.53	1,1,18	3,1,18	78.56
79.04	128		
80.94	1,0,22	1,1,22	80.96
		2,0,22	80.99
81.31	2,0,17		
		1,3,10	82.06
82.25	2,1,10		
		5,1,10	82.36
83.24	0,0,24	0,0,24	83.27
84.08	1,2,11		
		4,2,11	84.14
84.95	0,1,23	1,1,23	84.93
		330	85.03
85.15	300		
		600	85.35
			85.60^b^
		5,1,12	86.27
			87.50^b^
87.67	0,2,19		
		1,3,13	88.14
		336	88.19
88.30	306		
	2,1,13		
		4,2,13	88.35
		5,1,13	88.44
		606	88.50
		1,3,14	90.55
90.70	1,2,14		
		4,2,14	90.71
		5,1,14	90.80
91.11	2,0,20	2,2,20	91.07
		4,0,20	91.22
92.25	309	339	92.16
		609	92.47
93.37	1,0,25	1,1,25	93.38
95.97	2,1,16		
		5,1,16	96.11
96.70	0,0,27	0,0,27	96.74
97.86	0,1,26	1,1,26	97.88
98.49	0,2,22	2,2,22	98.48
		4,0,22	98.65
98.86	1,2,17		

a Calculated on the basis of a rhombohedral unit cell, $R\bar{3}$, *a* =3.9448(8) and *c* =27.8400(8) Å.

b Calculated on the basis of an orthorhombic unit cell, Cmmm, *a* =6.8188(3), *b* =3.9531(2), and *c* =27.830(1) Å.

c Apparently due to an unidentified superstructure.

**Table 7:** x-ray powder diffraction data for the *α*_1_′ phase (CaO:1/2Bi_2_O_3_ mol ratio 3:8, 780 °C quench, sample not ground)

*d* obs(Å)	Rel *I* (%)	2 *θ* obs	2 *θ* calc^a^	*hkl*
8.990	2	9.83		
4.669	4	18.99		
3.5296	7	25.21		
3.5050	6	25.39		
3.1565	100	28.25	28.26	003
2.9946	2	29.81		
2.9492	1	30.28		
2.7339	58	32.73	32.71	202
2.3510	4	38.25		
2.0031	5	45.23		
1.9517	3	46.49		
1.9341	54	46.94	46.96	024
1.8882	2	48.15		
1.8801	5	48.37		
1.7875	1	51.05		
1.7752	2	51.43		
1.6940	1	54.09		
1.6492	51	55.69	55.72	205
1.6184	1	56.84		
1.5799	5	58.36	58.35	042
1.5770	5	58.48	58.46	006
1.5666	1	58.90		
1.5482	2	59.67		
1.4401	1	64.67		
1.3906	2	67.27		
1.3680	6	68.54	68.55	404
1.3515	1	69.49		
1.3078	2	72.17		
1.2762	1	74.25		
1.2581	1	75.50		
1.2558	8	75.67	75.66	241
1.2537	8	75.82	75.80	027
1.2231	8	78.07	78.09	226
1.2089	1	79.16		
1.1828	1	81.27		
1.1796	1	81.53		
1.1528	1	83.85		
1.1174	5	87.16	87.15	600
1.1155	4	87.35	87.33	208
1.0533	5	94.00	94.03	425
1.0245	7	97.50		
1.0077	7	99.70		

a Calculated on the basis of a rhombohedral unit cell, $R\bar{3}$, *a* =7.7427(9) and c =9.465(1) Å.

**Table 8:** x-ray powder diffraction data for the *α*_1_*″* phase (CaO:1/2Bi_2_O_3_ mol ratio 3:8, 760 *°*C quench, not ground)

*d* obs (Å)	Rel *I* (%)	2 *θ* obs	2 *θ* calc^a^	*hkl*^a^	2 *θ* calc^b^	*hkl*^b^
8.812	<2	10.03	10.05	300	10.05	101
4.631	1	19.15	19.16	051	19.16	301
	<1	21.41	2:3		2:3	
3.5618	15	24.98	24.99	502	24.99	$10\bar{3}$
3.5120	11	25.34	25.35	701	25.35	$11\bar{1}$
3.2156	27	27.72	27.72	003	27.72	$40\bar{2}$
3.1208	100	28.58	28.58	081	28.58	$40\bar{2}$
	2	29.38	2:3		2:3	
3.0225	7	29.53	29.55	303	29.55	$30\bar{3}$
	1	30.80^c^				
	<1	31.09^c^				
	1	32.27^c^				
2.7226	55	32.87	32.87	802	32.87	004
	<1	34.39^c^				
	<1	34.57^c^				
2.5817	<1	34.72	34.73	381	34.73	113
2.3417	4	38.41	38.41	832	38.41	511
2.3265	3	38.67	38.65	0,11,1	38.66	503
2.3231	3	38.73	38.73	850	38.74	313
2.1934	2	41.12	41.12	054	41.12	701
2.1707	<1	41.57	41.56	244	d	
2.1485	1	42.02	42.00	514	42.04	105
2.0322	8	44.55	44.57	704	44.56	$51\bar{3}$
1.9866	4	45.63	45.64	13,0,1	45.65	305
1.9466	28	46.62	46.62	084	46.62	800
1.9039	34	47.73	47.73	880	47.73	020
8.812	<2	10.03	10.05	300	10.05	101
4.631	1	19.15	19.16	051	19.16	301
	<1	21.41	2:3		2:3	
1.9866	4	45.63	45.64	13,0,1	45.65	305
1.9466	28	46.62	46.62	084	46.62	800
1.9039	34	47.73	47.73	800	47.73	020
1.8828	9	48.30	48.29	853	48.30	115
					48.30	711
1.8382	<1	49.55	49.54	235	d	
1.8125	4	50.30	50.31	505	51.31	505
1.7929	3	50.89	50.90	384	50.90	$31\bar{5}$
1.7613	<1	51.87	51.88	13,3,1	51.89	315
					51.89	$32\bar{1}$
1.7176	4	53.29	53.29	075	53.29	713
1.7008	1	53.86	53.86	0,11,4	53.87	901
1.6786	4	54.63	54.62	3,13,2	54.62	$12\bar{3}$
1.6652	12	55.11	55.10	805	55.10	$40\bar{6}$
1.6384	34	56.09	56.09	883	56.09	422
1.6253	20	56.58	56.58	16,0,1	56.58	406
1.6102	4	57.16	57.14	11,5,3	57.14	$32\bar{3}$
					57.15	$52\bar{1}$
1.6079	4	57.25	57.25	006	57.25	$80\bar{4}$
1.5821	4	58.27	58.29	306	58.28	$70\bar{5}$
					58.29	$90\bar{3}$
1.5650	4	58.97	58.94	835	58.95	$91\bar{1}$
1.5602	13	59.17	59.17	0,16,2	59.17	024
					59.17	804
1.5526	4	59.49	59.48	13,0,4	59.48	$10\bar{7}$
1.5490	2	59.64	59.63	295	d	
1.5033	1	61.65	61.64	11,0,5	61.64	$30\bar{7}$
	<1	62.58^c^		*		
1.4738	<1	63.02	63.03	16,3,1	63.03	523
1.4382	1	64.77	64.78	13,3,4	64.78	$11\bar{7}$
					64.79	$72\bar{1}$
1.4303	1	65.17	65.18	16,1,3	65.18	913
1.4218	1	65.61	65.63	8,13,1	65.63	715
1.3773	3	68.01	68.01	13,8,2	68.01	317
1.3743	3	68.18	68.18	19,0,1	68.18	325
1.3614	6	68.92	68.93	16,0,4	68.93	008
					68.93	820
1.3338	1	70.55	70.56	18,0,3	70.57	$11,0,\bar{3}$
1.3221	4	71.27	71.27	856	71.27	$51\bar{7}$
					71.27	$11,1,\bar{1}$
1.3127	2	71.86	71.86	3,13,5	71.86	$52\bar{5}$
1.2942	2	73.05	73.04	707	73.03	$70\bar{7}$
					73.04	915
1.2717	2	74.56	74.55	11,11,3	74.56	$12,0,\bar{2}$
			74.57	087	74.57	$81\bar{6}$
1.2687	2	74.77	74.79	16,3,4	74.80	921
1.2536	4	75.83	75.83	0,16,5	75.84	12,0,2
1.2360	11	77.10	77.09	8,16,1	77.09	426
1.2285	4	77.66	77.66	886	77.66	$82\bar{4}$
1.2256	4	77.88	77.89	387	77.88	$71\bar{7}$
1.2168	3	78.55	78.56	11,5,6	78.56	$92\bar{3}$
1.2065	8	79.35	79.33	16,8,2	79.34	824
1.2011	2	79.78	79.78	10,15,1	79.79	$10,0,\bar{6}$
			79.79	208		
1.1798	1	81.52	81.51	3,16,5	81.51	$32\bar{7}$
1.1703	1	82.33	82.34	21,0,3	82.34	309
1.1526	2	83.87	83.88	13,8,5	83.87	$11\bar{9}$
1.1489	2	84.21	84.23	078	84.23	$11,1,\bar{5}$
1.1402	1	85.00	84.99	18,6,3	85.00	$23\bar{4}$
			85.00	13,0,7	85.00	$50\bar{9}$
					85.00	630
					85.00	$13,1,\bar{1}$
1.1331	2	85.66	85.67	16,1,6	85.66	$31\bar{9}$
					85.67	$52\bar{7}$
1.1272	1	86.22	86.23	0,19,5	86.24	11,2,1
					86.24	13,0,3
1.1226	1	86.66	86.69	11,13,4	86.69	$53\bar{3}$
1.1074	5	88.15	88.16	8,16,4	88.16	028
					88.16	$43\bar{4}$
					88.16	12,1,4
1.0990	4	88.96	88.97	24,0,0	88.98	808
1.0922	2	89.70	89.69	16,10,3	88.69	228
			89.70	13,3,7	89.69	$51\bar{9}$
					89.69	10,2,4
					89.70	11,2,3
1.0768	1	91.35	91.38	5,19,4	91.38	$33\bar{5}$
1.0641	1	92.75	92.76	309	92.75	$11,0,\bar{7}$
					92.75	$13,0,\bar{5}$
1.0575	1	93.51	93.51	16,0,7	93.50	$4,0,1\bar{0}$
					93.51	12,2,2
1.0468	3	94.76	94.74	16,8,5	94.74	832
					94.74	12,2,2
1.0402	4	95.55	95.56	24,0,3	95.56	4,0,10
			95.57	7,12,7	95.56	12,0,6
1.0343	1	96.28	96.27	2,12,8	d	
1.0212	<1	97.93	97.93	4,15,7	d	
1.0203	<1	98.05	98.06	639	d	
1.0115	1	99.20	99.22	21,8,1	99.22	719
1.0002	1	100.73	100.72	3,13,8	100.72	$15,1,\bar{1}$
0.9968	1	101.21	101.19	16,13,3	101.19	329
					101.20	11,2,5
0.9946	1	101.52	101.50	8,13,7	101.50	15,1,1
0.9898	1	102.20	102.20	26,1,1	102.18	15,0,3
0.9781	1	103.92	103.90	19,0,7	103.90	$52\bar{9}$
0.9733	2	104.64	104.64	859	104.64	$15,1,\bar{3}$
0.9622	1	106.37	106.37	0,2,10	106.36	$14,0,\bar{6}$
0.9520	2	108.03	108.02	16,16,0	108.03	828
					108.04	040
0.9432	1	109.51	109.52	5,25,1	109.54	16,1,0
0.9371	1	110.57	110.58	21,8,4	110.58	3,1,11
0.9332	1	111.26	111.27	26,2,3	111.29	$93\bar{5}$
0.9289	1	112.05	112.05	11,5,9	112.05	$11,2,\bar{7}$
0.9258	1	112.61	112.60	0,25,5	112.62	15,0,5
0.9242	1	112.91	112.91	2,7,10	112.92	2,3,
			112.92	2,24,5		
0.9127	2	115.13	115.11	3,20,7	115.11	12,2,6
0.9104	5	115.58	115.56	0,19,8	115.57	15,2,1
					115.57	17,0,1
0.9074	3	116.19	116.17	24,0,6	116.18	12,3,0
			116.21	9,13,8	116.18	16,0,4
0.8984	2	118.05	118.06	20,4,7	118.07	$12,3,\bar{2}$
0.8939	1	119.02	119.00	29,0,2	119.01	7,0,11
0.8780	1	122.64	122.66	29,2,0	d	
0.8755	1	123.25	123.23	5,24,5	123.23	1,2,11
					124.24	139
0.8738	1	123.66	123.66	13,11,8	123.66	$11,3,\bar{5}$
0.8732	1	123.80	123.81	21,13,2	123.79	139
					123.80	$74\bar{1}$
0.8710	1	124.35	124.37	5,18,8	d	
0.8665	1	125.49	125.49	27,6,0	d	

a Calculated on the basis of a rhombohedral unit cell, $R\bar{3}$, *a* =30.4640(5) and *c* =9.6477(2) Å.

b Calculated on the basis of a monoclinic unit cell, B2/m, *a* = 15.5819(3), *b* = 3.8077(1), *c* = 10.8955(3) Å, and *β* =91.829(2)°.

c Apparently due to an unidentified superstructure.

d Not indexable by the monoclinic all.

**Table 9:** x-ray powder diffraction data for the body centered cubic phase (CaO:1/2Bi_2_O_3_ mol ratio 9:10, 1000 °C quench)

*d* obs (Å)	Rel *I* (%)	2 *θ* obs	2 *θ* calc^a^	*hkl*
3.0006	100	29.75	29.73	110
2.1239	34	42.53	42.52	200
1.7330	51	52.78	52.77	211
1.5011	14	61.75	61.75	220
1.3430	12	70.00	70.02	310
1.2255	3	77.89	77.88	222
1.1346	10	85.52	85.51	321
1.0617	1	93.03	93.06	400
1.0008	3	100.65	100.66	330
0.9494	2	108.45	108.46	420
0.9052	1	116.64	116.63	332
0.8667	1	125.43	125.45	422
0.8326	2	135.39	135.37	510

a Calculated on the basis of a body centered cubic cell with *a* = 4.2458(1) Å.

**Table 10:** x-ray powder diffraction data for the distorted body centered cubic phase with line splitting and superstructure (CaO:1/2Bi_2_O_3_ mol ratio 2:3, 860 °C)

*d* obs (Å)	Rel *I* (%)	2 *θ* obs	2 *θ* calc^a^	*hkl*
8.699	1	10.16		
7.950	1	11.12		
7.783	1	11.36		
4.828	3	18.36		
4.635	1	19.13		
4.460	1	19.89		
4.2267	2	21.00		
4.1698	1	21.29		
4.0826	1	21.75		
3.9849	1	22.29		
3.8868	1	22.86		
3.5379	1	25.15		
3.4714	3	25.64		
3.3997	2	26.19		
3.3164	1	26.86		
3.2291	1	27.60		
3.1410	1	28.39		
3.0972	3	28.80		
3.0015	100	29.74	29.73	110
2.8841	4	30.98		
2.8245	2	31.65		
2.7801	1	32.17		
2.7526	1	32.50		
2.7184	1	32.92		
2.5924	1	34.57		
2.5467	1	35.21		
2.5300	1	35.45		
2.4859	1	36.10		
2.4609	1	36.48		
2.4143	1	37.21		
2.3901	1	37.60		
2.3218	1	38.75		
2.3012	1	39.11		
2.2861	2	39.38		
2.2800	2	39.49		
2.1621	4	41.74		
2.1233	23	42.54	42.52	200
2.0531	2	44.07		
2.0187	1	44.86		
1.9815	2	45.75		
1.9746	2	45.92		
1.9270	1	47.12		
1.8897	1	48.11		
1.8440	2	49.38		
1.8253	1	49.92		
1.8111	1	50.34		
1.7908	1	50.95		
1.7720	4	51.53		
1.7524	7	52.15		
1.7335	49	52.76	52.77	211
1.6990	3	53.92		
1.6871	2	54.33		
1.6673	3	55.03		
1.6626	3	55.20		
1.6502	1	55.65		
1.6252	1	56.28		
1.6078	1	57.25		
1.5278	1	60.55		
1.5111	3	61.29		
1.5025	9	61.68	61.75	220
1.4951	3	62.02		
1.3651	2	68.70		
1.3532	1	69.39		
1.3481	10	69.69	70.02	310
1.3356	2	70.44		
1.3235	1	71.18		

a Calculated on the basis of a body centered cubic cell with *a* =4.2458 (1) Å.

**Table 11:** X-ray powder diffraction data for the compound Ca_5_Bi_14_O_41_

*d* obs (Å)	Rel *I*(%)	2 *θ* obs	2 *θ* cale^a^	*hkl*
9.840	3	8.98	8.96	011
8.972	4	9.85	9.83	110
8.316	1	10.63	10.62	101
8.133	1	10.87	10.85	111
7.419	1	11.92	11.90	002
7.279	1	12.15	12.14	020
6.932	1	12.76	12.74	012
6.632	1	13.34	13.33	$\bar{1}11$
6.549	1	13.51	13.49	$\bar{1}11$
6.334	1	13.97	13.97	012
6.307	1	14.03	14.03	$\bar{12}1$
5.690	1	15.56	15.55	121
5.521	1	16.04	16.04	022
4.849	1	18.28	18.26	030
4.800	1	18.47	18.46	211
4.782	1	18.54	18.56	031
4.593	2	19.31	19.29	122
4.537	7	19.55	19.54	$01\bar{3}$
			19.55	$2\bar{1}1$
4.467	1	19.86	19.85	031
4.3143	3	20.57	20.58	$\bar{1}03$
4.2429	1	20.92	20.94	123
4.2150	1	21.06	21.08	122
4.1298	1	21.50	21.49	$11\bar{3}$
4.0736	2	21.80	21.81	222
3.9277	2	22.62	22.60	$21\bar{2}$
3.8620	2	23.01	22.99	230
3.7652	2	23.61	23.63	$2\bar{1}2$
3.6838	13	24.14	24.11	141
			24.16	$22\bar{0}$
3.6525	5	24.35	24.33	$22\bar{2}$
3.5576	11	25.01	24.99	203
3.5534	9	25.04	25.06	223
3.4903	16	25.50	25.51	142
3.4308	8	25.95	25.97	$1\bar{3}2$
3.4063	13	26.14	26.15	$10\bar{4}$
3.3336	2	26.72	26.71	$21\bar{3}$
3.3178	4	26.85	26.85	$\bar{2}22$
3.2997	8	27.00	27.00	310
3.2877	8	27.10	27.11	033
3.2293	75	27.60	27.61	$1\bar{3}3$
3.1347	100	28.45	28.47	$0\bar{4}2$
3.1272	72	28.52	28.53	034
3.1112	97	28.67	28.69	214
3.0744	94	29.02	29.02	$32\bar{1}$
3.0539	8	29.22	29.24	$12\bar{4}$
3.0195	9	29.56	29.55	$1\bar{4}2$
2.9743	7	30.02	30.01	$\bar{2}23$
2.9361	2	30.42	30.41	$3\bar{1}1$
2.9323	2	30.46	30.44	$2\bar{2}3$
			30.48	115
2.9285	2	30.50	30.51	$31\bar{2}$
2.9053	4	30.75	30.74	$\bar{2}32$
2.8662	2	31.18	31.17	025
2.8422	1	31.45	31.47	$2\bar{1}4$
2.8212	1	31.69	31.69	$3\bar{1}2$
2.7997	2	31.94	31.94	$05\bar{1}$
2.7777	2	32.20	32.19	$14\bar{3}$
17718	2	3127	3125	303
2.7250	96	32.84	32.84	$11\bar{5}$
			32.86	252
2.6971	50	33.19	33.21	$32\bar{1}$
2.6369	2	33.97	33.99	$\bar{3}03$
2.5976	2	34.50	34.52	$2\bar{2}4$
2.5766	2	34.79	34.79	253
2.5426	1	35.27	35.27	$\bar{3}13$
2.5371	1	35.35	35.36	$\bar{2}42$
2.4861	1	36.10	36.10	154
2.4584	2	36.52	36.54	420
2.4391	2	36.82	36.81	061
2.4276	3	37.00	36.99	$\bar{2}34$
			37.01	060
2.4057	6	37.35	37.34	261
2.4001	5	37.44	37.41	422
2.3964	4	37.50	37.48	$0\bar{1}6$
			37.51	$0\bar{5}3$
2.3523	3	38.23	38.24	$22\bar{5}$
2.3185	4	38.81	38.78	$3\bar{3}2$
2.3088	4	38.98	39.00	$43\bar{1}$
2.3008	3	39.12	39.11	216
2.2952	6	39.22	39.20	$2\bar{2}5$
2.2929	6	29.26	39.29	$4\bar{1}1$
2.2896	3	39.32	39.32	$\bar{1}26$
			39.32	423
2.2868	5	39.37	39.38	$25\bar{3}$

a Calculated on the basis of a triclinic cell, $P\bar{1}$, *a* =9.934(1), *b* = 15.034(2), *c* = 15.008(2) Å, *α* = 82.65(1), *β* =85.27(1), and *γ* = 77.17(1)°.

**Table 12:** X-ray powder diffraction data for the compound CaBi_2_O_4_ (CaO:l/2Bi_2_O_3_ 33:67)

*d* obs (Å)	Rel *I*(%)	2 *θ* obs	2 *θ* cale^a^	*hkl*	|*F*| cale
8.847	4	9.99	9.98	$11\bar{1}$	35
8.324	2	10.62	10.62	110	27
5.977	7	14.81	14.81	200	79
5.802	2	15.26	15.27	020	32
5.282	2	16.77	16.77	111	32
5.029	5	17.62	17.60	002	15
5.018	5	17.66	17.64	021	46
4.957	1	17.88	17.85	$31\bar{2}$	14
4.7413	6	18.70	18.70	$22\bar{1}$	56
4.4316	6	20.02	20.03	$22\bar{2}$	57
		21.44^2:3^			
3.8179	20	23.28	23.27	$11\bar{3}$	78
3.8018	31	23.38	23.38	022	130
3.7700	11	23.58	23.59	310	*19*
3.6808		24.16	24.18	130	
3.6029	2	24.69	24.68	112	31
3.4308	5	25.95	25.95	$40\bar{4}$	79
3.3546	9	26.55	26.53	$42\bar{2}$	58
3.3385	14	26.68	26.67	$31\bar{4}$	81
3.3312	15	26.74	26.77	$13\bar{2}$	78
3.3190	8	26.84	26.83	221	42
3.2723	42	27.23	27.24	$20\bar{4}$	247
3.2374	8	27.53	27.52	131	72
3.1941	22	27.91	27.89	$51\bar{3}$	117
3.1631	100	28.19	28.21	$33\bar{2}$	276
3.0859	12	28.91	28.92	$33\bar{1}$	78
3.0817	12	28.95	28.96	$42\bar{1}$	59
		29.33^2:3^			
2.9879	48	29.88	29.87	400	289
2.9503	16	30.27	30.25	$33\bar{3}$	87
			30.28	311	74
2.9053	2	30.75	30.76	023	14
2.8970	1	30.84	30.82	040	5
2.8502	2	31.36	31.67	$22\bar{4}$	43
2.8178	5	31.73	31.75	$11\bar{4}$	65
2.7853	14	32.11	32.10	041	16
2.7769	17	32.21	32.24	330	22
2.7470	30	32.57	32.57	$60\bar{4}$	234
2.7058	44	33.08	33.07	132	209
2.6705	5	33.53	33.53	$24\bar{2}$	46
			33.53	$51\bar{5}$	38
2.6559	2	33.72	33.71	420	31
2.6559	6	33.90	33.92	222	17
2.6422	5	33.96	33.97	$31\bar{5}$	18
2.6086	1	34.35	34.35	240	24
2.5882	1	34.63	34.61	$33\bar{4}$	18
2.5567	1	35.07	35.04	$60\bar{2}$	23
2.5198	18	35.60	35.58	$42\bar{5}$	106
2.5185	15	35.62	35.64	004	135
2.4821	1	36.16	36.15	$62\bar{4}$	22
2.4552	1	36.57	36.56	$53\bar{2}$	15
2.4494	1	36.66	36.63	$24\bar{3}$	19
2.4359	3	36.87	36.89	$53\bar{4}$	49
2.3933	1	37.55	37.53	331	41
2.3708	2	37.92	37.93	$44\bar{2}$	43
2.3618	2	38.07	38.06	312	28
2.3571	3	38.15	38.14	241	42
2.3411	6	38.42	38.42	$62\bar{2}$	77
2.3271	5	38.66	38.67	$71\bar{4}$	59
2.3014	1	39.11	39.11	$22\bar{5}$	23
2.2957	1	39.21	39.23	$40\bar{6}$	40
2.2857	2	39.39	39.67	$15\bar{1}$	32
2.2762	2	39.56	39.55	150	42
2.2669	2	39.73	39.73	$44\bar{1}$	46
2.2500	5	40.04	40.02	133	68
2.2377	6	40.27	40.27	$53\bar{5}$	91
2.2177	3	40.65	40.64	$33\bar{5}$	58
2.1934	2	41.12	41.11	043	50
2.1598	3	41.79	41.78	151	59
2.1393	5	42.21	42.21	114	75
2.1339	7	42.32	42.32	$42\bar{6}$	67
2.1272	10	42.46	42.46	$62\bar{6}$	104
2.1225	6	42.56	42.58	$62\bar{1}$	53
2.1130	5	42.76	42.77	$35\bar{1}$	81
2.0693	8	43.71	43.73	$35\bar{3}$	98
2.0466	9	44.22	44.21	332	118
		44.67^2:3^			
2.0236	2	44.75	44.75	$73\bar{4}$	26
2.0137	7	44.98	44.97	$44\bar{5}$	80
2.0112	6	45.04	45.02	$15\bar{3}$	63
2.0061	9	45.16	45.17	$80\bar{6}$	49
2.0049	12	45.19	45.20	350	90
1.9986	28	45.34	45.35	$53\bar{6}$	173
1.9936	17	45.46	45.49	600	134
1.9767	3	45.87	45.88	$73\bar{3}$	57
1.9526	8	46.47	46.46	$82\bar{5}$	98
1.9330	24	46.97	46.98	060	170
			46.98	$71\bar{7}$	126
1.9119	12	47.52	47.52	422	104
1.9100	13	47.57	47.58	$22\bar{6}$	100
1.8987	24	47.87	47.85	$33\bar{6}$	117
			47.89	061	117
1.8953	19	47.96	47.95	204	176
1.8650	33	48.79	48.79	$73\bar{2}$	231
1.8611	24	48.90	48.93	$26\bar{2}$	77
1.8459	1	49.33	49.32	351	29
1.8244	2	49.95	49.93	$11\bar{6}$	42
1.8217	2	50.03	50.00	$91\bar{5}$	35
1.8200	2	50.08	50.06	$42\bar{7}$	40
1.8105	2	50.36	50.36	243	52
		50.44^2:3^			
1.8038	5	50.56	50.54	062	66
1.7991	10	50.70	50.69	$44\bar{6}$	123
1.7805	8	51.27	51.25	$26\bar{3}$	109
1.7785	7	51.33	51.35	153	91
1.7610	3	51.88	51.89	333	54
1.7438	6	52.43	52.43	$60\bar{8}$	118
1.7282	6	52.94	52.94	$71\bar{8}$	103
1.7156	6	53.36	53.37	$80\bar{8}$	143
1.7067	5	53.66	53.66	$46\bar{1}$	102
1.6869	12	54.34	54.32	$91\bar{3}$	110
			54.34	$84\bar{5}$	109
1.6784	4	54.64	54.64	$84\bar{4}$	66
			54.65	006	48
1.6744	4	54.78	54.76	063	63
1.6640	11	55.15	55.15	$26\bar{4}$	116
1.6544	16	55.50	55.48	$93\bar{6}$	164
1.6503	15	55.65	55.62	314	108
			55.68	$84\bar{6}$	95
1.6413	4	55.98	55.95	640	70
1.6357	5	56.19	56.18	408	71
			56.20	$91\bar{8}$	60
1.6335	5	56.27	56.29	$75\bar{3}$	66
1.6319	6	56.33	56.35	$93\bar{4}$	66
1.6227	10	56.68	56.67	460	146
1.6118	5	57.10	57.09	026	110
1.6023	1	57.47	57.46	$93\bar{7}$	38
1.5959	3	57.72	57.73	171	57
1.5874	18	58.06	58.06	532	139
			58.07	154	82
1.5834	12	58.22	58.21	$75\bar{6}$	43
1.5807	12	58.33	58.33	$66\bar{4}$	130
			58.35	$66\bar{3}$	87
1.5704	2	58.75	58.77	$91\bar{2}$	37
1.5595	1	59.20	59.20	$53\bar{8}$	30
1.5573	1	59.29	59.28	$37\bar{3}$	42
1.5425	6	59.92	59.93	$66\bar{2}$	95
1.5343	7	60.27	60.25	$31\bar{8}$	117
1.5330	8	60.33	60.33	064	82
1.5311	6	60.41	60.43	$26\bar{5}$	73
1.5188	2	60.95	60.94	$93\bar{8}$	58
1.5150	4	61.12	61.12	461	84
1.5055	3	61.55	61.58	353	81
1.4969	5	61.94	61.93	$75\bar{7}$	85
1.4941	8	62.07	62.06	800	134
			62.08	11,1, $\bar{7}$	131
1.4851	4	62.49	62.52	263	75
1.4836	4	6256	62.58	404	153
1.4793	8	62.76	62.76	$62\bar{9}$	89
1.4753	8	62.95	62.97	622	93
1.4715	5	63.13	63.12	$51\bar{9}$	65
1.4692	4	63.24	63.24	$57\bar{2}$	66
1.4649	4	63.45	63.46	$57\bar{4}$	78
1.4606	4	63.66	63.65	$24\bar{7}$	51
			63.68	11,1, $\bar{8}$	61
1.4520	6	64.08	64.06	046	110
			64.10	$20\bar{8}$	136
1.4414	3	64.61	64.60	$73\bar{9}$	42
1.4374	5	64.81	64.83	$84\bar{1}$	78
1.4346	6	64.95	64.94	081	116
1.4299	8	65.19	65.18	10,2, $\bar{9}$	103
1.4295	8	65.21	65.24	$35\bar{7}$	105
1.4216	3	65.62	65.61	$95\bar{4}$	80
1.4170	6	65.86	65.88	136	130
1.4090	1	66.28	66.28	$86\bar{4}$	28
			66.29	533	25
1.4027	1	66.62	66.61	10,0, $\bar{2}$	37
			66.63	$95\bar{7}$	39
1.4010	2	66.72	66.71	11,1, $\bar{9}$	67
1.3984	2	66.85	66.86	462	58
1.3977	3	66.89	66.91	$26\bar{6}$	57
1.3942	3	67.08	67.07	065	65
1.3923	3	67.18	67.21	$86\bar{6}$	91
1.3820	1	67.75	67.73	$28\bar{3}$	49
1.3802	1	67.85	67.85	11,3, $\bar{5}$	42
1.3750	5	68.14	68.13	750	84
1.3736	6	68.22	68.23	$55\bar{8}$	73
			68.23	12,0, $\bar{8}$	138
1.3657	2	68.67	68.67	372	63
1.3631	2	68.82	68.80	10,2, $\bar{2}$	63
1.3614	3	68.92	68.92	$46\bar{7}$	43
			68.92	$93\bar{1}$	37
1.3610	3	68.94	68.95	8,2, $\bar{10}$	56
1.3540	5	69.35	69.34	354	82
			69.38	264	78
1.3465	4	69.79	69.79	$48\bar{1}$	69
1.3457	5	69.84	69.83	$86\bar{7}$	68
			69.85	$95\bar{8}$	67

a Calculated on the basis of a monoclinic unit cell, space group C2/*c*, *a* = 16.6295(8), *b* = 11.5966(5), *c* = 14.0055(6) Å, and *β* = 134.036(3)°.

**Table 13:** X-ray powder diffraction data for the compound Ca_4_Bi_6_O_13_

*d* obs (Å)	Rel *I* (%)	2 *θ* obs	2 *θ* cale^a^	*hkl*	|*F*| calc
8.708	13	10.15	10.17	200	250
5.629	4	15.73	15.75	110	136
4.434	1	20.01	19.99	111	45
4.346	5	20.42	20.42	400	217
4.145	47	21.42	21.40	310	571
3.614	52	24.61	24.60	002	138
3.338	52	26.68	26.69	202	118
3.0386	100	29.37	29.35	112	748
2.9987	68	29.77	29.75	510	893
2.9694	31	30.07	30.05	020	829
2.8117	8	31.80	31.81	220	306
2.7794	44	32.18	32.18	402	766
2.7250	2	32.84	32.84	312	93
2.4519	2	36.62	36.61	420	187
2.4107	1	37.27	37.28	003	103
2.3225	1	38.74	38.74	203	116
			38.74	421	45
2.3088	3	38.98	38.98	512	158
2.2952	5	39.22	39.22	022	263
2.2918	3	39.28	39.90	710	185
2.2609	12	39.84	39.85	602	501
2.2187	3	40.63	40.62	222	165
2.1717	13	41.55	41.54	800	667
2.0847	1	43.37	43.39	313	85
2.0815	1	43.44	43.46	801	35
2.0733	1	43.62	43.61	620	64
2.0291	53	44.62	44.61	422	846
1.9686	1	46.07	46.09	130	159
1.9357	4	46.90	46.92	712	227
1.8744	7	48.53	48.54	330	437
1.8625	1	48.86	48.87	802	182
1.8368	2	49.59	49.60	910	189
1.8288	1	49.82	49.79	223	81
1.8078	14	50.44	50.44	004	917
1.7991	12	50.70	50.71	622	466
1.7699	2	51.60	51.60	204	195
1.7537	11	52.11	52.11	820	602
1.7376	9	52.63	52.62	10,0,0	679
1.7285	23	52.93	52.93	132	607
1.7206	18	53.19	53.18	114	141
			53.18	530	769
1.6688	1	54.98	54.97	404	197
1.6640	2	55.15	55.16	332	149
1.6574	10	55.39	55.40	314	423
1.6373	31	56.13	56.13	912	804
1.5782	1	58.43	58.45	822	170
1.5670	1	58.89	58.92	10,0,2	93
1.5533	2	59.46	59.44	532	183
1.5486	21	59.66	59.67	514	696
			59.67	730	142
1.5446	10	59.83	59.84	024	675
1.5265	1	60.61	60.59	11,1,0	141
1.5206	3	60.87	60.88	224	232
1.5004	7	61.78	61.79	10,2,0	599
1.4857	5	62.46	62.47	040	682
1.4645	2	63.47	63.48	240	332
1.4552	1	63.92	63.93	424	185
1.4462	1	64.37	64.37	005	112
1.4262	1	65.38	65.37	205	120
1.4233	3	65.53	65.53	732	321
1.4064	2	66.42	66.41	11,1,2	221
1.4060	2	66.44	66.46	440	235
1.3896	6	67.33	67.33	804	528
1.3831	1	67.69	67.71	930	197
1.3738	1	68.21	68.20	042	210
1.3574	1	69.15	69.16	242	168
1.3445	3	69.91	69.91	12,0,2	406
1.3314	1	70.70	70.71	134	150
1.3102	10	72.02	72.03	442	593
1.3041	6	72.41	72.40	13,1,0	618
1.3008	9	72.62	72.61	334	355
1.2914	9	73.24	73.24	932	615
1.2739	1	74.41	74.43	10,2,3	67
1.2588	5	75.46	75.47	824	490
1.2530	3	75.87	75.88	10,0,4	545
1.2466	9	76.33	76.34	534	619
1.2418	4	76.68	76.71	642	375
1.2354	1	77.15	77.17	11,3,0	142
1.2261	4	77.84	77.83	840	506
1.2249	3	77.93	77.94	12,2,2	344
1.1856	1	81.04	81.04	150	162
1.1783	5	81.65	81.64	116	472
1.1740	4	82.01	82.00	14,0,2	615
1.1690	2	82.44	82.46	11,3,2	239
1.1643	2	82.84	82.86	350	411
1.1614	3	83.10	83.11	406	527
			83.11	842	176
1.1544	4	83.71	83.71	10,2,4	494
1.1476	3	84.32	84.32	044	565
1.1378	3	85.22	85.22	244	260
1.1374	3	85.26	85.28	15,1,0	386
1.1291	2	86.04	86.03	10,4,0	466
1.1265	4	86.28	86.28	152	455
1.1243	2	86.49	86.48	550	501
1.1168	1	87.22	87.23	026	213
1.1126	1	87.63	87.63	606	323
1.1082	3	88.07	88.08	13,3,0	566
			88.08	352	149
1.0983	1	89.07	89.08	934	191
1.0918	6	89.74	89.73	14,2,2	579
1.0863	1	90.32	90.33	16,0,0	167
1.0817	5	90.82	90.83	426	551
1.0736	1	91.70	91.68	552	220
1.0578	4	93.47	93.48	13,1,4	520
1.0420	1	95.34	95.34	626	303
1.0370	1	95.94	95.95	12,4,0	154
1.0277	6	97.10	97.10	136	415
1.0200	1	98.08	98.06	16,2,0	245
1.0148	3	98.77	98.77	844	426
1.0074	4	99.75	99.73	916	556
1.0001	1	100.75	100.75	15,3,0	332
0.9968	2	101.21	101.21	12,4,2	353
0.9903	1	102.13	102.13	060	478
0.9841	1	103.03	103.05	260	254
0.9787	2	103.82	103.82	354	342
0.9745	6	104.45	104.44	952	496
0.9704	3	105.08	105.06	17,1,2	405
0.9655	2	105.85	105.84	18,0,0	557
0.9627	2	106.28	106.31	15,1,4	322
0.9576	2	107.10	107.09	10,4,4	396
0.9549	3	107.55	107.56	554	431
0.9446	3	109.27	109.25	13,3,4	484
0.9327	3	111.35	111.33	462	420
0.9263	1	112.52	112.51	12,0,6	275
0.9210	3	113.52	113.50	14,4,2	473
0.9182	3	114.05	114.04	18,2,0	516
0.9148	3	114.71	114.70	446	433

a Calculated on the basis of an orthorhombic unit cell, space group C2rara, *a* = 17.3795(5), *b* =5.9419(2), and *c* =7.2306(2) Å.

**Table 14:** X-ray powder diffraction data for the compound Ca_2_Bi_2_O_5_

*d* obs (Å)	Rel *I* (%)	2 *θ* obs	2 *θ* cale^a^	*hkl*	|*F*| cale
9.461	4	9.34	9.36	100	59
8.717	7	10.14	10.12	001	56
			10.16	$0\bar{1}1$	63
8.001	11	11.05	11.07	$\bar{1}01$	109
7.303	4	12.11	12.14	$\bar{1}10$	66
6.916	4	12.79	12.81	$11\bar{1}$	70
5.069	4	17.48	17.49	$11\bar{2}$	40
			17.49	$0\bar{2}1$	71
5.013	2	17.68	17.69	$0\bar{1}2$	56
4.965	1	17.85	17.87	$\bar{2}01$	31
4.721	4	18.78	18.78	200	98
4.648	16	19.08	19.09	$\bar{1}02$	191
4.421	6	20.07	20.09	020	93
4.352	3	20.39	20.41	$0\bar{2}2$	79
4.237	8	20.95	20.97	$\bar{2}11$	141
4.182	19	21.23	21.23	$12\bar{2}$	206
3.9940	10	22.24	22.24	$\bar{2}02$	146
3.9746	7	22.35	23.33	$1\bar{1}2$	104
			22.42	111^b^	120
3.9209	7	22.66	22.65	$2\bar{1}1$	130
3.8341	11	23.18	23.19	210	169
3.7480	1	23.72	23.69	$1\bar{2}2$	38
3.7065	4	23.99	24.00	120	103
3.5243	3	25.25	25.23	$2\bar{2}1$	81
3.5120	3	25.34	25.33	102	82
3.4957	6	25.46	25.48	$\bar{1}21$	127
3.4530	2	25.78	25.79	$22\bar{2}$	65
3.3834	2	26.32	26.31	$22\bar{1}$	37
3.3696	3	26.43	26.43	$\bar{3}01$	98
3.3571	3	26.53	26.52	$12\bar{3}$	76
3.3361	14	26.70	26.69	021	197
3.3226	8	26.81	26.80	$\bar{2}12$	88
			26.83	012	115
3.3045	11	26.96	26.96	$0\bar{3}2$	192
3.2806	6	27.16	27.13	$0\bar{1}3$	135
			27.18	023	60
3.2501	5	27.42	27.41	$1\bar{3}1$	133
3.2179	4	27.70	27.70	$\bar{2}21$	117
3.1456	8	28.35	28.33	300	127
			28.35	$13\bar{2}$	102
3.1059	54	28.72	28.74	$31\bar{1}$	471
3.0600	3	29.12	29.15	$22\bar{3}$	97
3.0006	100	29.75	29.73	$\bar{2}03$	427
			29.74	$\bar{1}30$	410
			29.77	211	116
2.9938	57	29.82	29.82	$2\bar{2}$	469
2.9099	5	30.70	30.69	003	132
2.8989	9	30.82	30.82	$0\bar{3}3$	111
2.8897	36	30.92	30.93	$1\bar{2}3$	83
			30.93	$13\bar{3}$	380
			31.09	$\bar{3}20$^b^	122
2.8361	36	31.52	31.49	$\bar{3}12$	228
			31.53	112	391
			31.55	$3\bar{1}1$	82
2.7828	7	32.14	32.13	$\bar{2}30$	172
			32.17	310	99
2.7519	2	32.51	32.50	202	81
2.7234	5	32.86	32.84	$3\bar{2}1$	155
2.6620	3	33.64	33.63	$\bar{3}03$	101
2.6049	1	34.40	34.38	$32\bar{3}$	68
2.5510	12	35.15	35.14	$11\bar{4}$	259
2.5336	4	35.40	35.40	$22\bar{4}$	91
			35.41	$0\bar{4}2$	95
2.5171	2	35.64	35.64	$21\bar{4}$	68
			35.65	$\bar{4}01$	72
2.5055	1	35.81	35.83	$0\bar{2}4$	86
2.4761	2	36.25	36.24	$\bar{2}31$	117
2.4500	1	36.65	36.63	$1\bar{4}1$	73
2.4333	2	36.91	36.92	013	106
2.4182	2	37.15	37.13	$41\bar{2}$	84
2.4132	3	37.23	37.24	$0\bar{1}4$	122
2.3921	5	37.57	37.55	$3\bar{2}2$	177
2.3805	1	37.76	37.75	$\bar{3}13$	79
2.3594	4	38.11	38.09	400	144
2.3271	2	38.66	38.64	$31\bar{4}$	93
2.3116	6	38.93	38.95	$33\bar{2}$	107
2.3071	10	39.01	38.99	$32\bar{4}$	138
			39.02	$2\bar{4}1$	245
2.3003	9	39.13	39.14	$41\bar{3}$	139
			39.15	$\bar{4}20$	133
2.2896	12	39.32	39.31	$14\bar{1}$	259
2.2857	11	39.39	39.39	230	158
2.2757	13	39.57	39.56	$\bar{2}21$	348
2.2658	4	39.75	39.76	$1\bar{2}4$	163
2.2484	1	40.07	40.06	$3\bar{3}2$	55
2.2192	1	40.62	40.61	$1\bar{3}4$	69
2.2083	4	40.83	40.83	$24\bar{3}$	116
2.1722	2	41.54	41.55	$33\bar{1}$	94
2.1578	3	41.83	41.84	203	146
2.1311	3	42.38	42.40	$\bar{1}23$	146
2.1055	2	42.92	42.95	$2\bar{4}3$	85
2.0765	1	43.55	43.55	$3\bar{4}1$	96
2.0558	24	44.01	44.00	$24\bar{1}$	400
			44.01	$1\bar{4}4$	207
2.0536	24	44.06	44.06	140	140
2.0326	3	44.54	44.54	$23\bar{5}$	149
			44.54	$4\bar{3}1$	198
2.0206	4	44.82	44.81	$\bar{1}141$	39
2.0167	7	44.91	44.90	$0\bar{5}2$	159
2.0154	5	44.94	44.95	502	149
			44.95	$\bar{3}32$	137
2.0087	2	45.10	45.10	321	123
1.9911	18	45.52	45.50	$0\bar{2}5$	396
1.9841	4	45.69	45.72	$32\bar{5}$	180
1.9763	4	45.88	45.87	$34\bar{3}$	168
1.9722	6	45.98	45.96	$43\bar{3}$	202
			46.11	$\bar{5}12$^b^	117
1.9490	2	46.56	46.54	330	103
1.9322	3	46.99	46.98	$4\bar{1}2$	123
1.9287	4	47.08	47.05	$\bar{5}03$	104
			47.10	041	158
			47.12	$51\bar{2}3$^b^	109
1.9081	3	47.62	47.64	$\bar{5}21$	164
1.9043	3	47.72	47.74	$43\bar{4}$	95
1.8946	3	47.98	47.99	$15\bar{4}$	124
1.8850	1	48.24	48.26	$\bar{3}41$	124
1.8462	5	49.32	49.34	$\bar{4}14$	204
			49.82	$\bar{3}05$^b^	102
1.8261	7	49.90	49.90	$42\bar{5}$	266
1.8112	6	50.34	50.36	$51\bar{4}$	270
1.7939	13	50.86	50.87	$5\bar{1}1$	396
1.7743	16	51.46	51.46	$\bar{2}24$	368
			51.47	$\bar{5}30$	266
1.7660	6	51.72	51.73	$3\bar{5}2$	280
1.7569	3	52.01	52.01	$\bar{3}33$	176
1.7459	3	52.36	52.36	$52\bar{1}$	156
1.7418	2	52.49	52.47	$44\bar{3}$	107
1.7388	4	52.59	52.58	$0\bar{5}5$	100
1.7349	8	52.72	52.73	$\bar{1}42$	261
1.7306	3	52.86	52.88	$\bar{4}05$	127
1.7267	7	52.99	53.01	$44\bar{4}$	300
1.7200	4	53.21	53.23	$2\bar{5}4$	177
			53.26	322^b^	109
			53.38	$4\bar{3}3$^b^	145
1.7135	4	53.43	53.45	$25\bar{5}$	223
1.7017	12	53.83	53.84	421	377
			54.12	$3\bar{5}3$^b^	104
			54.12	$35\bar{3}$^b^	118
1.6883	9	54.29	54.27	$33\bar{6}$	250
			54.28	$0\bar{6}3$	248
1.6846	4	54.42	54.40	$\bar{2}15$	108
			54.41	$14\bar{6}$	128
			54.62	232^b^	113
			54.66	$4\bar{1}3$^b^	151
1.6738	7	54.80	54.78	$1\bar{6}3$	269
			54.95	$1\bar{6}2$^b^	142
1.6682	16	55.00	54.98	$1\bar{5}5$	305
			54.99	042	242
			54.99	$\bar{6}11$	117
			55.06	$21\bar{6}$^b^	213
			55.07	412^b^	128
1.6643	15	55.14	55.13	$\bar{4}33$	183
			55.14	$3\bar{1}4$	386
1.6596	3	55.31	55.32	223	128
1.6530	5	55.55	55.55	$\bar{6}03$	260
1.6503	5	55.65	55.63	$\bar{1}15$	217
			55.95	$61\bar{2}$^b^	101
			56.06	$0\bar{4}6$^b^	147
1.6370	4	56.14	56.14	$11\bar{6}$	105
1.6357	5	56.19	56.18	$\bar{1}51$	231
1.6248	1	56.60	56.58	$2\bar{6}2$	110
			56.71	$4\bar{5}1$^b^	119
1.6201	2	56.78	56.77	$1\bar{6}4$	106
1.6172	2	56.89	56.89	105	161
1.6092	7	57.20	57.21	$\bar{4}42$	313
1.5997	4	57.57	57.56	$4\bar{5}2$	242
1.5919	2	57.88	57.87	$53\bar{5}$	154
1.5884	2	58.02	58.00	$61\bar{1}$	150
1.5846	2	58.17	58.16	$2\bar{6}1$	152
1.5797	2	58.37	58.35	133	266
			58.74	$53\bar{1}$^b^	111
1.5694	1	58.79	58.81	$13\bar{6}$	129
1.5672	2	58.88	58.88	$16\bar{5}$	145
1.5621	1	59.09	59.11	$2\bar{5}5$	133
1.5523	6	59.50	59.51	$62\bar{2}$	300
1.5453	2	59.80	59.82	124	159

a Calculated on the basis of a triclinic unit cell, space group $P\bar{1}$, *a* = 10.1222(7), *b* = 10.1466(6), *c* = 10.4833(7) Å, *α* = 116.912(5), *β* = 107.135(6), and *γ* = 92.939(6)°.

b Calculated |*F*| greater than 100 but cannot be distinguished from nearby peaks.

**Table 15:** X-ray powder diffraction data for the “C-mon” Metastable Phase

*d* obs (Å)	Rel *I* (%)	2 *θ* obs	2 *θ* cale^a^	*hkl*
12.405	2	7.12	7.15	001
10.419	3	8.48	8.51	200
9.009	6	9.81	9.83	$20\bar{1}$
7.219	1	12.25	12.27	201
5.221	4	16.97	16.99	$40\bar{1}$
4.865	11	18.22	18.24	202
4.489	27	19.76	19.74	$40\bar{2}$
4.447	4	19.95	19.94	401
	1	20.62^b^		
4.109	2	21.61	21.59	$11\bar{1}$
3.7049	4	24.00	24.00	310
	5	24.07^b^		
3.6718	8	24.22	24.23	$31\bar{1}$
3.6044	11	24,68	24.69	402
			24.69	$11\bar{2}$
3.5730	6	24.90	24.93	203
3.4491	22	25.81	25.79	112
3.4360	23	25.91	25.88	311
3.3583	11	26.52	26.53	$31\bar{2}$
3.3521	11	26.57	26.57	$60\bar{2}$
3.1576	2	28.24	28.24	601
3.1565	2	28.25	28.24	$20\bar{4}$
3.0922	3	28.85	28.87	004
3.0457	74	29.30	29.31	$51\bar{1}$
3.0406	88	29.35	29.34	$11\bar{3}$
3.0265	97	29.49	29.51	312
3.0056	100	29.70	29.59	510
			29.60	$60\bar{3}$
2.9267	69	30.52	30.50	403
2.8299	2	31.59	31.61	511
2.7989	11	31.95	31.96	204
	4	32.17^CaO^		
2.6605	6	33.66	33.64	$80\bar{1}$
2.6101	2	34.33	34.35	$60\bar{4}$
2.6042	1	34.41	34.38	313
2.5976	1	34.50	34.52	800
2.5336	5	35.40	35.40	$20\bar{5}$
2.4715	6	36.32	36.31	005
2.4359	9	36.87	36.88	801
	9	37.34^CaO^		
2.3541	2	38.20	38.19	$51\bar{4}$
2.3036	1	39.07	39.07	$71\bar{3}$
2.2463	2	40.11	40.10	$80\bar{4}$
			40.11	314
2.2234	3	40.54	40.51	802
2.1919	25	41.15	41.13	020
2.1593	5	41.80	41.80	021
2.1534	6	41.92	41.93	712
2.1470	5	42.05	42.08	220
2.1225	7	42.56	42.58	$11\bar{4}$
2.1102	29	42.82	42.80	$20\bar{6}$
2.0783	25	43.51	43.51	$40\bar{6}$
2.0760	27	43.56	43.54	10,0,0
2.0630	17	43.85	43.86	405
2.0223	3	44.78	44.79	$42\bar{1}$

a Calculated on the basis of a monoclinic unit cell, C2/m, *a* =21.295(4), *b* =4.3863(8), *c* = 12.671(2) Å, and *β* = 102.74(1)°.
